# Impaired Response Inhibition in the Rat 5 Choice Continuous Performance Task during Protracted Abstinence from Chronic Alcohol Consumption

**DOI:** 10.1371/journal.pone.0109948

**Published:** 2014-10-15

**Authors:** Cristina Irimia, Roxanne N. Tuong, Tammy Quach, Loren H. Parsons

**Affiliations:** Committee on the Neurobiology of Addictive Disorders, The Scripps Research Institute, La Jolla, California, United States of America; University of Chicago, United States of America

## Abstract

Impaired cognitive processing is a hallmark of addiction. In particular, deficits in inhibitory control can propel continued drug use despite adverse consequences. Clinical evidence shows that detoxified alcoholics exhibit poor inhibitory control in the Continuous Performance Task (CPT) and related tests of motor impulsivity. Animal models may provide important insight into the neural mechanisms underlying this consequence of chronic alcohol exposure though pre-clinical investigations of behavioral inhibition during alcohol abstinence are sparse. The present study employed the rat 5 Choice-Continuous Performance Task (5C-CPT), a novel pre-clinical variant of the CPT, to evaluate attentional capacity and impulse control over the course of protracted abstinence from chronic intermittent alcohol consumption. In tests conducted with familiar 5C-CPT conditions EtOH-exposed rats exhibited impaired attentional capacity during the first hours of abstinence and impaired behavioral restraint (increased false alarms) during the first 5d of abstinence that dissipated thereafter. Subsequent tests employing visual distractors that increase the cognitive load of the task revealed significant increases in impulsive action (premature responses) at 3 and 5 weeks of abstinence, and the emergence of impaired behavioral restraint (increased false alarms) at 7 weeks of abstinence. Collectively, these findings demonstrate the emergence of increased impulsive action in alcohol-dependent rats during protracted alcohol abstinence and suggest the 5C-CPT with visual distractors may provide a viable behavioral platform for characterizing the neurobiological substrates underlying impaired behavioral inhibition resulting from chronic intermittent alcohol exposure.

## Introduction

Alcohol dependence is associated with significant disruptions in executive functions [1], [2] which likely contribute to the loss of control and relapse that characterize alcohol addiction [3]. In particular, diminished capacity to restrain impulsive behavior is an important predictor of relapse [4], and impaired decision making and a reluctance to seek help [5] reinforces the addiction cycle.

Impaired executive function is commonly conceptualized to result in increased impulsivity [6]–[9], although other cognitive deficits such as working-memory impairments have also been documented in alcoholics [2]. Impulsivity is broadly defined as “behavior performed without appropriate forethought” [10], and in the context of alcoholism it may result in behavioral inflexibility and insensitivity to the negative outcomes associated with decision-making, as well as decreased control over the ability of alcohol cues to bias attention or elicit behaviors [11].

Behavioral consequences of increased impulsivity have been operationalized as a lack of attention, a preference for immediate small rewards versus larger delayed rewards, and deficits in suppressing motor responses [12], [13]. Alcoholics exhibit increased impulsivity while performing tasks such as the Continuous Performance Task (CPT) [14]–[17], Go/NoGo [18] and Stop Signal Reaction Time Task (SSRTT) [15], [19], [20] that probe different aspects of behavioral inhibition. In particular, CPT procedures are used to measure sustained attention, vigilance and impulsivity, and require subjects to respond to target (Go) stimuli and inhibit responses to non-target (NoGo) stimuli both of which are repeatedly presented in close succession [21]. Responding following NoGo stimuli (commission errors) provides an index of the capacity to restrain prepotent motor responses [22]. In alcoholics cognitive impairments are particularly evident under conditions of high cognitive load [23]–[25]. For example, CPT performance by alcoholics is often indistinguishable from controls under standard task conditions, though alcoholics exhibit slower and less accurate performance than controls when the task requires the suppression of irrelevant prepotent information [24].

Few attempts have been made to model the effect of chronic alcohol exposure on impulse control in a preclinical setting, although such models are essential to conduct mechanistic studies and screening of pharmacological treatments. Despite commonly employed protocols for chronic alcohol exposure in rodents [26] and the existence of rat versions of the SSRT and Go/NoGo tasks [22] there are no published reports on the effects of chronic alcohol exposure on the performance of these tasks. Three studies have employed the 5-Choice Serial Reaction Time Task (5-CSRTT) to probe for altered impulse control and attentional capacity following chronic alcohol exposure [27]–[29]. Two of these reports demonstrate that rats and mice previously exposed to chronic alcohol exhibit excessive premature responding (e.g. responses made prior to Go stimulus presentation) for at least 4 weeks following cessation of alcohol exposure [27], [28]. Because premature responding in this task is commonly used to index motor impulsivity [22], [30], [31] these findings provide evidence of persistent disruptions in impulse control following long-term alcohol exposure. However, the 5-CSRTT does not incorporate NoGo trials and in this respect these studies do not fully generalize to clinical research studies reporting deficits in the capacity to inhibit prepotent motor responses by alcoholics.

To address this the present experiments employed a recently developed rodent version of the CPT, the 5 Choice-CPT (5C-CPT) [32], [33], that incorporates random presentation of NoGo trials throughout a succession of rapidly presented Go trials. This approach offers several advantages. First, the 5C-CPT has high face validity with the human version of the task in that the Go and NoGo targets share common features (e.g. letters or numbers in the human task, different subsets of LED visual stimuli in the rodent task) that increase the task difficulty. Secondly, the 5C-CPT allows the evaluation of chronic alcohol effects on two different types of impulsive behaviors; the inability to wait for a stimulus presentation (impulsive action; indexed by premature responses) and the inability to restrain a prepotent response when presented with a NoGo stimulus (impaired behavioral inhibition or behavioral restraint; indexed by false alarm responses). These two facets of motor impulsivity are encoded by distinct but overlapping circuits in the brain [22], [34]–[36] and are influenced by different neurotransmitter systems [36]. Humans performing a reverse-translated version of the 5C-CPT activate brain areas known to be important for response inhibition [35]. Thirdly, the 5C-CPT provides indices suitable for the application of signal detection theory (SDT), thereby providing metrics of rodent behavior that are similar to those generated during cognitive profiling in persons with alcohol use disorder [14], children with ADHD and schizophrenics [21].

The present experiments tested the hypothesis that chronic intermittent alcohol exposure results in both increased impulsive action and impaired behavioral restraint that emerges and persists during protracted abstinence. Toward this goal separate groups of rats were trained in the 5C-CPT, given chronic intermittent access to either an EtOH-containing or EtOH-free liquid diet, then further probed in the 5C-CPT during abstinence periods ranging from 3 hours to 7 weeks.

## Materials and Methods

### Subjects

Male Wistar rats (Charles River, Wilmington, MA, USA) weighing 225–250 g at the beginning of the experiments were housed 2 per cage in a humidity and temperature-controlled (22°C) vivarium on a 12 h light/dark cycle (lights off at 10AM). Throughout the experiment with the exception of the liquid diet exposure described below, the animals were maintained at 90% of their free feeding body weight to maintain motivation to respond for food during 5C-CPT testing.

### Ethics statement

All procedures were conducted in strict adherence to the NIH Guide for Care and Use of Laboratory Animals. The protocol was approved by the Institute Animal Care and Use Committee of The Scripps Research Institute (permit# 120023).

### Apparatus

CPT training and testing occurred in 6 identical five-hole nose-poke operant chambers (Med Associates Inc., St. Albans, VT) enclosed in ventilated sound-attenuating chambers. Data collection was achieved using Med-PC v4.0 (Med Associates). One wall of the box had an array of five response holes, and the opposite wall had a magazine connected to a food hopper. The dispenser delivered food pellets (45 mg, grain-based rodent tablet-formerly manufactured as Formula PJAI by Research Diets& PJ Noyes-Test diet, Richmond, NJ) to the magazine. The boxes were fitted with cue lights (denoted in this paper as Stimulus lights) and a white house light was located above the food magazine. A fan in the sound-attenuating box provided ventilation and helped mask inadvertent background noises.

### 5C-CPT behavioral procedure

Each 5C-CPT session consisted of 120 trials, 70% Go and 30% NoGo trials. During the Go trials, a brief visual stimulus (2 sec) appeared in one of the five apertures signaling the correct response location. A nose-poke response in the lit aperture either during stimulus presentation or within an additional 2 sec limited hold (LH) period was counted as a correct response and was rewarded with a food pellet. During the NoGo trials, the visual stimulus appeared in all the five apertures concomitantly for 2 sec. Withholding response during the NoGo stimulus presentation and 2 sec LH was counted as a correct rejection and was rewarded with a food pellet. Collection of the food pellet after any correct trial initiated a variable intertrial interval (ITI: 3, 5, 9 or 11 sec) waiting period, after which the next trial began. Error responses included nose-pokes in non-target apertures during the Go trial (incorrect response), failure to withhold responding during the NoGo stimulus presentation (false alarm response), failure to withhold responding during the ITI (premature response) and failure to respond in any aperture during a trial (omission), and each of these errors was punished by withholding of food reward and a brief period of darkness (time out (TO), 5 sec). Additional nose-pokes during the TO were counted as perseverative responses and were punished by restarting the TO counter. The session ended when all the 120 trials were completed or more than 45 min elapsed from the beginning of the session. The parameters used to evaluate task performance are described in Table 1. The change in each parameter during the session was interpreted in conjunction with all the other recorded indices.

**Table 1:** Behavioral indices used to evaluate CPT performance.

Measure	Definition	Description
p[CorrectResponse]	correct/(correct + incorrect) responses	index of response accuracy
Latency to correct response	time lapsed between stimulus presentation and a correct response (sec)	index of decision time to make a correct response
p[Omissions]	omissions/(omission + correct + incorrect) responses	index of ability or motivation to perform the task
Latency to feeder	time to collect the earned food pellet from the magazine (sec)	index of motivation to perform the task
p[Hit Rate]	correct/(correct + incorrect + omission) responses	index of attentional capacity
p[FalseAlarm]	false alarms/(false alarm + correct rejection) responses	index of impulsive action
Latency to False Alarms	time elapsed between non-target stimulus presentation and a false alarm response	index of decision time to make an incorrect (false alarm) response
Sensitivity index (SI)	(p[HR]-p[FA])/(2*(p[HR]+p[FA])-(p[HR]+p[FA])^2^)	non-parametric index of the ability to discriminate between target and non-target trials [32]. Values range from -1 to +1, with +1 indicating that all the target and non-target trials were properly attended.
Responsivity Index RI) (bias)	(p[HR]+p[FA]-1)/(1-(p[FA]-p[HR])^2^)	non-parametric index of the willingness to respond to stimuli [32]. More positive values indicate a more liberal response strategy while more negative values indicate a conservative response strategy.
Premature responses	responses made during the inter-trial interval (ITI)	index of impulsive action
Perseverative responses	responses made during the time out (TO)	index of behavioral disinhibition

### Training schedule

Rats needed approximately 6 months of training to establish stable baseline performance on the 5C-CPT task. Initially, the animals were trained to perform 5-CSRTT using published procedures [28], [31]. When the rats responded with more than 80% accuracy to a Go stimulus presented for 2 sec, using a fixed intertrial interval of 5 sec, the task was modified to include an intertrial interval that was varied pseudo-randomly between 3, 5, 9 and 11 sec. Once the rats successfully acquired this version of the task (accuracy>80%, omissions<20%), the CPT training started using procedures previously described [33], [37]. Briefly, the Go and NoGo trials were initially presented in equal numbers during a session. For each rat individually, when the animal responded with>65% correct rejections using the formula (correct rejections*100/(correct rejections + false alarms)), the percentage of NoGo trials during future sessions was dropped to 40%, and then 30%. The initial stimulus duration was 8 sec, and as the percentage of NoGo trials was dropped during the task acquisition, the stimulus duration was also reduced to 4 sec, and then to 2 sec. 5C-CPT training was completed when rats responded with>80% accuracy on Go trials and>65% correct rejections on NoGo trials (false alarm rate <35%), when stimulus duration was 2 sec.

### 5C-CPT Challenge sessions

To evaluate the effects of prior EtOH exposure on 5C-CPT performance under more cognitively demanding conditions rats were given a series of “challenge” tests in which a visual distractor light was illuminated in tandem with presentation of the Go and NoGo stimuli. Three challenge conditions were evaluated, each distinguished by a different placement of the LED distractor relative to the 5C-CPT stimulus panel, in addition to a challenge session in which the house light was used as a distractor.

### Distractor 1: LED distractor not associated with stimulus panel

A small LED light was placed half way between the five-aperture panel where stimuli were presented and the food magazine, 2″ away from the metal grid. The LED light (ENV-321M, Med Associates Inc., St. Albans, VT) was taped outside of the transparent box wall. The LED was connected to the computer through the MedAssociates interface. During the session, the computer turned the LED on during every trial when a Go or a NoGo stimulus was presented. The distractor turned off when the rat made a nose-poke response, or after 2 sec (the length of the stimulus duration). The LED was not on during the intertrial interval. While turned on, the LED light emitted a small white light similar to the light presented in the five stimulus holes.

### Distractor 2: LED distractor directly adjacent to stimulus panel

In this condition the LED distractor light was located more closely to the five-apertures panel. The LED was placed 2″ above the metal grid, 0.5″ away from the left-side wall (which contained the curved wall and the five-holes panel) and 10″ from the right-side wall (the walls of the behavioral box were 10.5″×10.5″). The LED turned on during every trial while a Go or NoGo stimulus was presented. The distractor was turned off when the rat made a nose-poke response or after 2 sec (the length of the stimulus duration). The LED was not on during the intertrial interval.

### Distractor 3: LED distractor included in the stimulus panel

As described above, the 5C-CPT sessions were conducted in operant boxes with a curved left wall that had a panel of five apertures equally spaced where Go and NoGo stimuli were presented during the sessions. During this challenge test, neither Go nor NoGo stimuli were presented in the 3^rd^ (middle) aperture. Rather, whenever a light stimulus was presented in the adjacent apertures (or in all apertures in the case of NoGo trials), the LED in the middle aperture flashed with a frequency of 10 Hz. The intensity and color of the light was similar to the stimulus lights presented in the other 4 apertures. The distractor LED continued to flash until the end of the stimulus presentation in the other apertures or until the rat made a nose-poke response, whichever came first.

### House light distractor challenge test (negative control)

During this test the house light was programmed to flash on/off with a 10 Hz frequency during the session whenever the house light would normally be lit.

### Alcohol exposure

A liquid diet paradigm was employed for alcohol exposure as described in our previous publications [28]. The diet consisted of chocolate liquid nutritional supplement (Boost, Nestle S.A., Vevey, Switzerland) fortified with vitamins and minerals. The EtOH group received liquid diet supplemented with 10% (w/v) EtOH and the CON group received an EtOH-free diet supplemented with sucrose to equalize the caloric intake of both groups. The EtOH diet was supplemented with non-caloric sweetener to minimize taste differences between diets. All animals were given fresh diet daily, 2 h after the beginning of the dark cycle. Rats in the EtOH group each received 100 ml of diet daily, and the volume consumed was measured to the nearest ml using a volumetric graduated cylinder. Alcohol consumption was indexed by calculating the daily intake in g EtOH/kg/rat and blood alcohol levels (BALs) were determined once a week. For this approximately 100 µl of blood was collected in a microtube kept on ice. Samples were immediately centrifuged at 2000 g for 10 min, serum was decanted and assayed for EtOH content using the Alcohol Oxidase method (Analox Instrument LTD, Lunenburg, MA, USA) as previously described [28]. To normalize intake across groups the volume of diet available to the CON rats was regulated according the volume of diet consumed by partner rats in the EtOH group on the prior day (e.g. pair feeding).

### Experimental design

**Figure 1:**
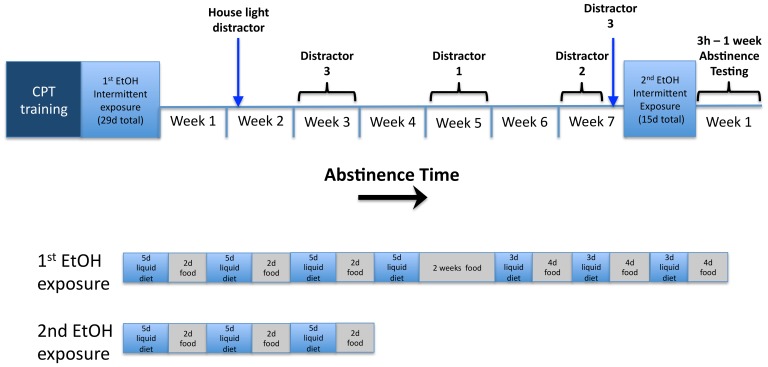
Experimental design. Following 5C-CPT training, animals were given intermittent access to either an EtOH-containing (10% w/v) or EtOH-free control liquid diet over the course of 9 weeks (“1^st^ EtOH exposure”, detailed in bottom panel; total of 29 days on liquid diet; average daily EtOH intake of 8.73±0.2 g/kg/day resulting in 210±14 mg% blood alcohol levels). 5C-CPT evaluations were performed during a subsequent 7-week abstinence period with visual distractor “challenge” tests performed during the 3^rd^, 5^th^ and 7^th^ week of abstinence to increase task difficulty in an effort to unveil EtOH-related cognitive impairment (see text for details of each distractor condition). EtOH-related disruptions in 5C-CPT performance during the first week of abstinence (3h – 5d) were evaluated following a second regimen of liquid diet exposure (“2^nd^ EtOH exposure”, detailed in bottom panel; 15d intermittent access over 3 weeks; average daily EtOH intake of 8.83±0.7 g/kg/day resulting in 289±12 mg% blood alcohol levels).

The temporal progression of testing is depicted in **Figure 1**. Following establishment of stable baseline 5C-CPT performance, rats were separated into two groups that did not differ in task performance and were given intermittent access to either control liquid diet (CON group, n = 17 rats) or liquid diet with 10% alcohol (w/v; EtOH group, n = 16) following procedures previously described [28]. Based on clinical and preclinical evidence demonstrating a positive correlation between the number of detoxification episodes and the prevalence of cognitive impairment [16], [28] a liquid diet regimen was devised to first establish robust EtOH intake (4 cycles of 5d liquid diet and 2d standard chow), followed by 2 weeks of abstinence and a subsequent 3 week period in which rats received repeating cycles of 3d of liquid diet and 4d of standard chow (totaling 29d exposure to liquid diet interspersed with periods of abstinence). 5C-CPT evaluations were performed during a subsequent 7-week abstinence period. Tests with standard 5C-CPT parameters were conducted 5d/week. During this time, rats were also presented with challenge sessions employing the previously described visual distractor conditions. Based on evidence that indices of motor impulsivity emerge within 7d abstinence and persist for at least 5 weeks of abstinence [28] these challenge tests were presented following 3 weeks (distractor condition 3), 5 weeks (distractor condition 1) and 7 weeks abstinence (distractor condition 2). To characterize group differences in adaptation to each distractor condition, the same challenge was presented in consecutive sessions until group differences in 5C-CPT performance dissipated (up to 5 consecutive sessions). A single session with distractor condition 3 (the first challenge presented) was given at the end of the 7-week abstinence period to evaluate the persistence of behavioral disruptions evident under this condition. Following a second regimen of liquid diet exposure (15d intermittent access over 3 weeks, consisting each week of 5d of liquid diet and 2d of standard chow) disruptions in 5C-CPT performance at different abstinence time points (3h–5d) were evaluated.

### Statistical analysis

**Figure 2:**
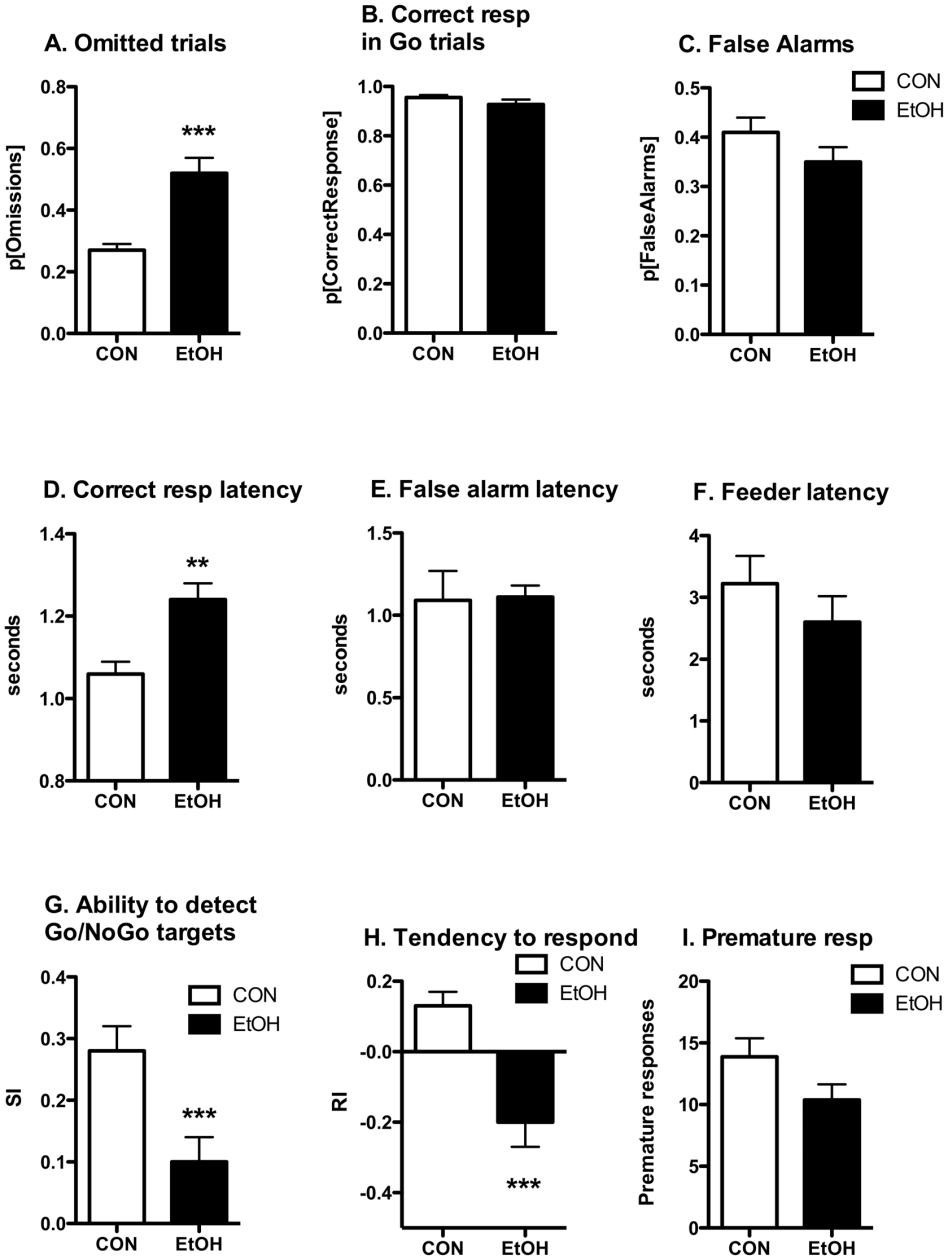
Impaired 5C-CPT performance during initial hours of EtOH abstinence. In a test performed 3 h into EtOH abstinence using familiar task conditions EtOH-exposed rats (n = 16) failed to respond to nearly half of the presented trials (**panel A**), took longer to elicit correct responses in Go trials (**panel D**), exhibited diminished discrimination between Go and NoGo trials (**panel G**) and a diminished tendency to respond (**panel H**) as compared with EtOH-naïve CON animals (n = 17), suggesting an impairment in the ability to perform the task. The lack of group differences in the percentage of correct responses in Go trials (**panel B**), false alarm latency (**panel E**) or the latency to retrieve the food rewards (**F**) suggests an equal capacity to “solve” the 5C-CPT task and a similar motivation to do so. There were no group differences in motor impulsivity (premature responses, **I**) or behavioral restraint (false alarms, **panel C**) at this abstinence time. Significant group effects denoted by * p<0.05, ** p<0.01, *** p<0.001 as determined by 1-way ANOVA.

**Figure 3:**
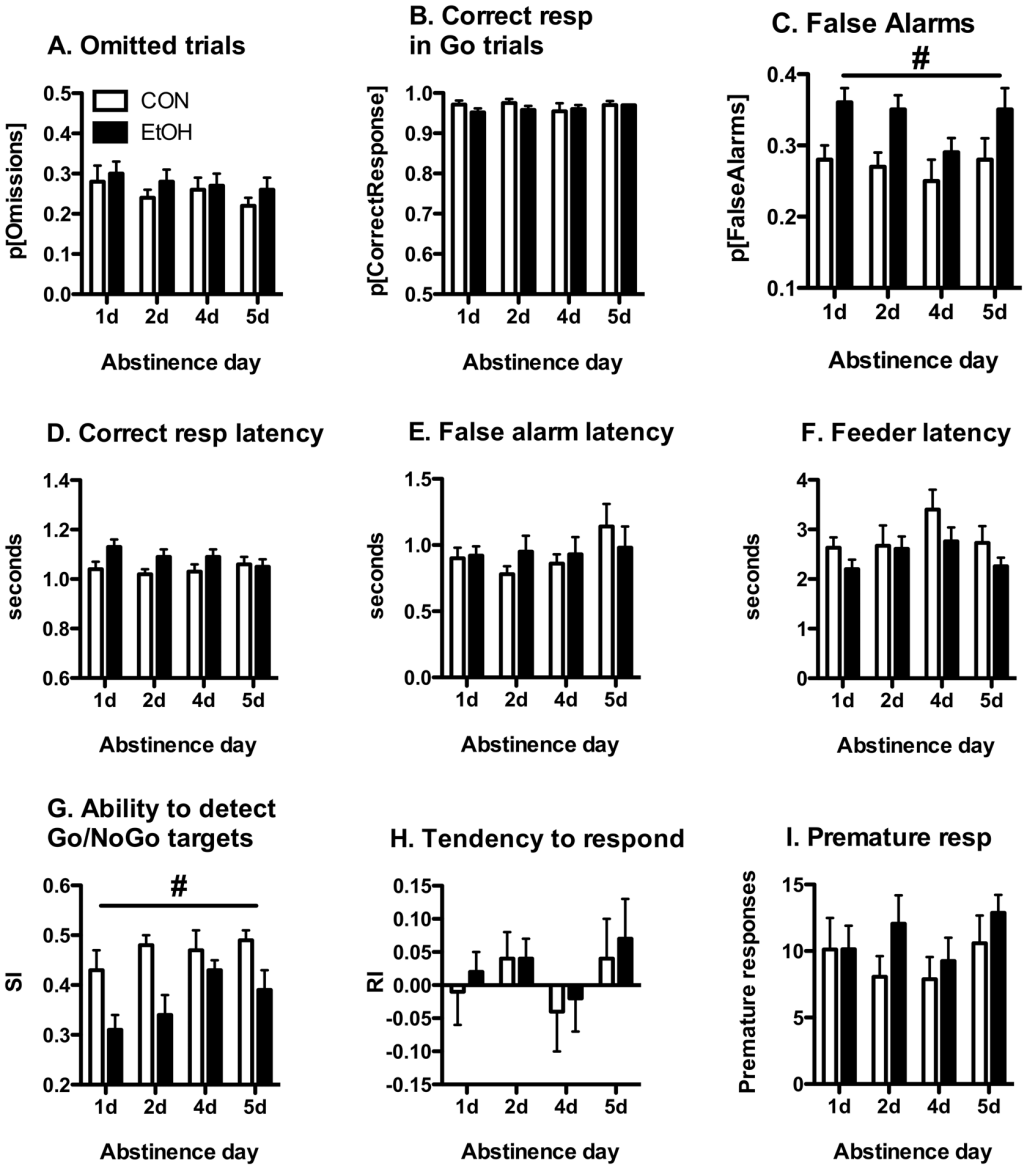
Increased false alarm errors during the first week of abstinence from chronic intermittent EtOH. When tested using familiar 5C-CPT parameters following 1–5 days of abstinence, EtOH-exposed rats (n = 16) elicited significantly more incorrect responses in NoGo trials (false alarm errors, **panel C**) and exhibited diminished discrimination between Go and NoGo trials (sensitivity index, **panel G**) as compared with EtOH-naïve control rats (n = 17). During this time there were no group differences in response accuracy (**panel B**), correct response latency (**panel D**), incorrect response latency (**panel E**), tendency to respond (**panel H**), premature responses (**panel I**), latency to retrieve the food rewards (**panel F**) or omissions (**panel A**). # denotes significant group effects based on repeated-measures ANOVA (p<0.05).

All data were analyzed using the PSAW Statistics package (SPSS, v18.0). Because all rats completed all trials in each session throughout the experiment the data were not normalized to a percentage of possible responses. The dependent variables for our analyses of 5C-CPT performance included accuracy, false alarm errors, omissions, correct response latency, feeder latency, response bias, target sensitivity, premature and perseverative responses. We examined the effects of acute withdrawal on 5C-CPT performance (**Figure 2**) using a 1-way ANOVA with Group (CON, EtOH) as between subjects factor for each task parameter independently. A 2-way ANOVA was used to evaluate the effects of alcohol exposure on task performance during the first week of abstinence (**Figure 3**) with Group (CON, EtOH) as the between subjects factor and time (d1- d5 of abstinence) as the within subjects factor. To evaluate the persistence of alcohol-related effects on 5C-CPT performance under standard task parameters during protracted abstinence (Table 2), we performed a repeated measures ANOVA with group (EtOH, CON) as the between-subjects factor and abstinence time points (as defined in Table 2) as the within-subjects factor.

**Table 2:** CPT performance during standard sessions following repeated cycles of liquid diet.

Measure	Group	Baseline following task acquisition (pre-EtOH exposure) (2d average)	First diet exposure d4-d5 abstinence (2d average)	First diet exposure 7 weeks abstinence (2d average)	Second diet exposure d4-d5 abstinence (2d average)
Accuracy	CON	96.87±0.36	96.78±0.49	96.53±0.68	96.26±1.08
(p[CorrectResp]*100)	EtOH	96.16±0.69	95.77±0.48	94.41±0.73	96.50±0.50
Latency to correct	CON	1.05±0.03	1.03±0.02	1.03±0.02	1.05±0.02
response (sec)	EtOH	1.08±0.02	1.05±0.02	1.07±0.02	1.07±0.03
Premature	CON	10.31±0.99	12.76±1.76	11.21±1.02	9.24±1.64
	EtOH	12.83±1.63	12.53±1.64	14.19±2.25	11.06±1.46
p[False Alarm]	CON	0.27±0.02	0.28±0.02	0.34±0.02	0.27±0.02
	EtOH	0.25±0.02	0.26±0.02	0.36±0.02	0.32±0.03
RI	CON	−0.08±0.04	0.02±0.03	0.07±0.04	0.00±0.05
	EtOH	−0.11±0.05	−0.10±0.05	0.03±0.04	0.03±0.05
SI	CON	0.41±0.03	0.46±0.03	0.39±0.03	0.48±0.03
	EtOH	0.43±0.02	0.41±0.03	0.33±0.03	0.41±0.03
Perseverative	CON	4.14±0.60	3.94±0.61	3.74±0.67	2.56±0.51
	EtOH	4.33±0.50	3.44±0.40	4.81±0.76	3.28±0.61
p[Omissions]	CON	0.31±0.02	0.24±0.02	0.26±0.02	0.24±0.02
	EtOH	0.31±0.02	0.31±0.03	0.28±0.02	0.26±0.03
Feeder	CON	2.50±0.24	2.96±0.31	2.60±0.24	3.06±0.32
Latency (sec)	EtOH	2.31±0.21	2.45±0.25	2.19±0.22	2.51±0.20

**Figure 4:**
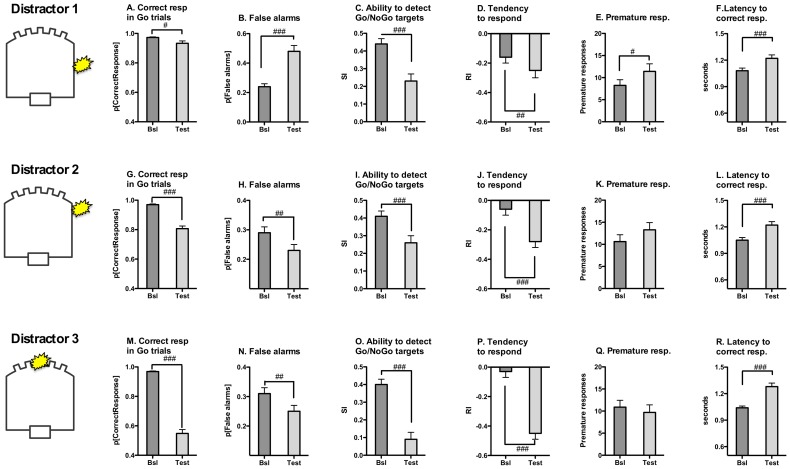
Characterization of the effects of visual distractors on 5C-CPT performance by EtOH-naïve control rats. In an effort to increase the cognitive load of the 5C-CPT a series of challenge tests were performed in which irrelevant prepotent visual distractors (LEDs) were illuminated in tandem with presentation of the Go and NoGo visual stimuli. Three challenge conditions were evaluated, each distinguished by a different placement of the LED distractor relative to the 5C-CPT stimulus panel (see text for details). Data shown are from control rats (n = 17) to demonstrate the effects of each distractor condition on 5C-CPT performance. All distractors reduced response accuracy (**panels A, G, M**) and increased the latency to correct response (**panels F, L, R**), indicative of increased cognitive load. In the presence of distractors rats were less able to discriminate between Go and NoGo signals (**panels C, I, O**) and chose a conservative response strategy (**panels D, J, P**). With exception of the latency to correct response, the disruption in each of these behavioral indices was progressively more pronounced upon presentation of distractor conditions 1, 2 and 3, respectively (see text for details). False alarm errors and premature responding were increased by distractor condition 1 (**panels B, E**), while distractor conditions 2 & 3 decreased false alarm errors (**panels H, N**) without disrupting premature responding (**panels K, Q**). Relative effects of each distractor condition on 5C-CPT performance was evaluated by within-subject ANOVA comparison of baseline performance under familiar task conditions (dark grey bars) and performance during the distractor challenge test (light grey bars). Significant effects are denoted by # p<0.05; ## p<0.01; ### p<0.001 (1-way ANOVA).

**Figure 5:**
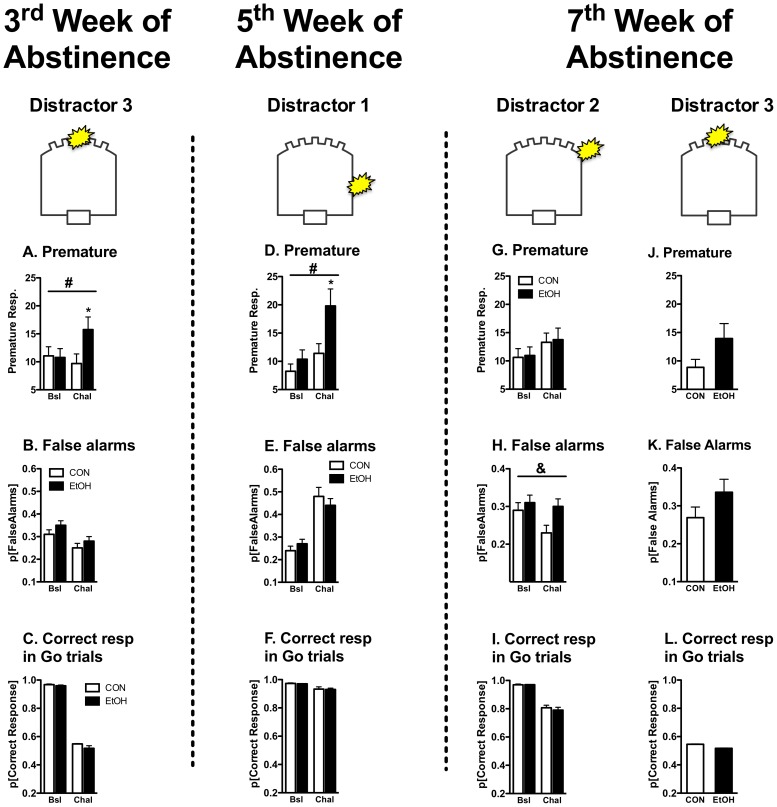
Evaluation of motor impulsivity and impaired behavioral restraint during distractor challenge tests over the course of 7 weeks of abstinence. Challenge tests with visual distractors were performed during the 3^rd^, 5^th^ and 7^th^ week of abstinence to characterize the temporal profile of altered 5C-CPT performance during protracted EtOH abstinence. Distinct challenge conditions were presented at each abstinence time to minimize habituation to the visual distractor. However, the initially presented distractor condition was presented again at the end of the 7^th^ abstinence week to probe the persistence of initially observed performance disruptions. Effects of each challenge condition were determined by comparing performance during challenge tests (Chal) with baseline performance under familiar conditions (BSL). Data from EtOH-exposed rats (n = 16) are black bars, data from EtOH-naïve controls (n = 17) are white bars. During the 3^rd^ and 5^th^ abstinence week EtOH-exposed rats exhibited increased motor impulsivity (premature responses; **panels A & D**) without disruption of behavioral restraint (false alarms; **panels B & E**). EtOH-related increases in motor impulsivity were not evident during the 7^th^ week of abstinence (**panel G**) (even under the initially employed distractor condition (**panel J**)) though EtOH-exposed rats exhibited significant impairment in behavioral restraint at this time (**panel H**). No group differences in response accuracy were evident at any abstinence time (**panels C, F, I & L**). **#** denotes significant group x challenge interaction and **&** denotes significant group main effects (p<0.05; Test vs. BSL ANOVA). * denotes significant group differences determined by Fisher's protected LSD (p<0.05).

The effects of distractor challenges on 5C-CPT performance by CON rats (**Figure 4**) were probed using 1-way ANOVA with test condition (baseline (the average of two sessions immediately prior to distractor test), distractor challenge (first presentation)) as the within-subjects factor. The comparative difficulty of each distractor condition was evaluated using repeated measures ANOVA with test condition (baseline, distractor challenge) and distractor type (conditions 1, 2, 3 and house light) as within-subjects factors. Separate analyses were performed for the EtOH and CON groups. Possible group differences in 5C-CPT performance during each distractor challenge test (**Figure 5**) were explored using 2-way mixed factorial ANOVA with group (EtOH, CON) as the between-subjects factor and test condition (baseline, distractor test) as the within-subjects factor. Possible group differences in the response to repeated challenges with a given distractor were evaluated by repeated measures ANOVA with group (EtOH, CON) as the between-subjects factor and number of sessions a given distractor condition was presented as the within-subjects factor. In the case of significant interactions in the mixed models, simple effects ANOVAs and Fisher's protected LSD test were used to further probe group differences. Statistical details germane to major conclusions of this report are presented in the Results section below, though all statistical results are presented in the Supplemental Information. All data used in the analyses described above are available in the Data S1 file.

## Results

### Task acquisition and EtOH exposure

Rats required 117 sessions to reliably acquire the 5C-CPT and establish stable baseline performance (Table 2, 3^rd^ column). Following training animals were separated into two groups that did not differ on baseline performance indices (Table 2, 3^rd^ column, p>0.1, NS). During the first and second periods of liquid diet exposure rats in the EtOH group consumed 8.73±0.20 g/kg/day and 8.83±0.68 g/kg/day EtOH, resulting in BALs of 209.75±14 and 289±12 mg%, respectively.

### 5C-CPT performance during acute abstinence from chronic intermittent EtOH exposure

Rats in the EtOH group demonstrated significant impairments in 5C-CPT performance during a test session conducted 3 h after the end of the 2^nd^ liquid diet exposure (**Figure 2**). Animals in the EtOH group failed to respond to almost half the trials (**Figure 2A**, omitted trials, F_1,30_ = 22.604, p<0.0001). The lack of group differences in the percentage of correct responses for the completed trials or the latency to retrieve the earned food rewards (**Figure 2B, 2F**, Table S1) suggests that both groups were able to “solve” the task and were similarly motivated to do so. Rather, increases in the time required by EtOH animals to make a correct response (**Figure 2D**, F_1,30_ = 12.088, p<0.01) coupled with diminished discrimination between Go and NoGo trials (**Figure 2G**, Sensitivity Index, F_1,30_ = 9.101, p<0.01) and a diminished tendency to respond (**Figure 2H**, Bias F_1,30_ = 16.732, p<0.001) suggest impaired information processing as a possible explanation for the increased number of missed trials by EtOH-exposed animals. However, the effects of somatic withdrawal or lingering intoxication on behavioral performance at this time-point cannot be ruled out. There were no group differences in false alarms or premature responses at this time point (**Figure 2C, 2I**, Table S1).

Significant group differences in the response to Go and NoGo trials were maintained during subsequent testing days (**Figure 3**), as evidenced by greater levels of inappropriate responding during NoGo trials (**Figure 3C**, False Alarms, group: F_1,31_ = 5.712, p<0.05; time: F_3,93_ = 3.238, p<0.05; time x group F_3,93_<1, NS) and diminished discrimination between Go and NoGo trials (**Figure 3G**; SI, group: F_1,31_ = 8.818, p<0.05; time: F_3,93_ = 5.461, p<0.01; time x group: F_3,93_ = 1.924, NS) by the EtOH-exposed animals. Similar to the animals' behavior at the 3 h withdrawal time-point, there were no group differences in response accuracy, feeder latency or premature responses (**Figure 3**, Table S2). Group differences in the number of omitted trials and the time required to make a correct response that were observed at the 3 h withdrawal time-point resolved during this period of early abstinence (**Figure 3**, Table S2).

CON rats exhibited significant differences in 5C-CPT performance in the sessions conducted at 3 h and 1d following cessation of liquid diet access: relative to the 3 h abstinence session performance at 1d abstinence was characterized by reduced false alarm errors (F_1,16_ = 25.265, p<0.0001), reduced response bias (session: F_1,16_ = 6.754, p<0.05) and increased target sensitivity (session: F_1,16_ = 20.083, p<0.001). These improved performance indices during the 2^nd^ post-diet test did not vary over the subsequent 3 test sessions (e.g. 1d-5d into abstinence; false alarm errors and response bias, SI, session: F_3,48_<1.693, NS). This suggests that task performance by the CON group rapidly improved to stable levels within the first two 5C-CPT sessions following a prolonged absence of training (9 weeks of intermittent liquid diet exposure). In contrast, task performance by EtOH rats did not significantly improve over the course of the first 5d of 5C-CPT testing following liquid diet exposure. This may reflect impaired re-acquisition of task performance following a prolonged absence of training, an effect that may contribute to the group differences in 5C-CPT performance during early abstinence.

### 5C-CPT performance during prolonged abstinence from chronic intermittent EtOH exposure

#### Performance with familiar testing conditions

By the 4^th^ day of abstinence, CON and EtOH rats were performing the 5C-CPT task at similar levels, and indistinguishable group performance was evident in 5C-CPT tests with standard task parameters conducted through 7 weeks of abstinence from liquid diet (Table 2). The two groups did not differ on measures of selective attention, decision time, behavioral disinhibition, sensitivity, response strategy, motivation and ability to attend to the task (accuracy, latency to correct response, false alarms, premature responses, perseverative responses, SI, bias, feeder latency, omissions, NS, Table S3).

#### Performance during distractor challenge tests

Because clinical evidence suggests that impaired cognitive performance and neuronal function in alcoholics is most evident under novel and challenging task conditions [23], we sought to increase the 5C-CPT difficulty by incorporating novel distractors during the session. In an effort to probe the temporal profile of group differences in cognitive ability during prolonged EtOH abstinence, distinct distractor conditions were presented following different periods of liquid diet abstinence (**Figure 1**). The relative impact of each distractor condition on 5C-CPT performance in CON animals is described below, followed by descriptions of the effect of distractor challenges on 5C-CPT performance in CON and ETOH rats following 3, 5 and 7 weeks of abstinence from liquid diet exposure.

*Distractor Condition 1*: Presentation of an LED light distractor in the middle of the wall separating the food magazine and the stimulus display panel during the 5C-CPT session resulted in moderate disruptions in task performance by CON rats as compared with performance in the absence of the distractor LED (**Figure 4** **A-F**; Table S4). Response accuracy during the Go trials decreased slightly (distractor challenge: F_1,16_ = 6.957, p<0.05) and the time required to make a correct response increased (F_1,16_ = 19.316, p<0.0001), indicative of increased attentional demands. In the context of stimulus ambiguity, CON rats were less able to discriminate between Go and NoGo trials (SI, distractor challenge: F_1,16_ = 25.638, p<0.0001) and chose a conservative response strategy (RI, test: F_1,16_ = 16.254, p<0.001) in the presence of the distractor. CON rats demonstrated increased behavioral disinhibition during the challenge test, making more false alarm errors (distractor challenge: F_1,16_ = 26.492, p<0.0001) and more premature responses (distractor challenge: F_1,16_ = 5.111, p<0.05). The motivation to perform the task did not change during the test (feeder latency, distractor challenge: F_1,16_ = 1.390, NS). This distractor condition induced a nearly identical pattern of performance disruptions in the EtOH group (**Figure S1 A–F**, Table S5), though the magnitude of effects differed between treatment groups (see below).

*Distractor Condition 2*: Presentation of an LED distractor directly adjacent to the panel where the stimulus lights were displayed resulted in more pronounced disruptions in task performance by CON rats (**Figure 4**, **G–L**; Table S4). Increased task difficulty manifested in deceased response accuracy during the Go trials (distractor challenge: F_1,16_ = 95.628, p<0.0001) and an increase in the time required to make a correct response (F_1,16_ = 27.070, p<0.0001). In the presence of this distractor rats were less able to discriminate between Go and NoGo trials (SI, distractor challenge: F_1,16_ = 28.492, p<0.0001), chose a conservative response strategy (RI distractor challenge: F_1,16_ = 23.810, p<0.0001) and made fewer false alarm errors (distractor challenge: F_1,16_ = 8.811, p<0.01). This distractor condition did not alter premature responses (distractor challenge: F_1,16_ = 2.602, NS) or the motivation to perform the task (feeder latency, distractor challenge: F_1,28_ = 1.60, NS). This distractor condition induced a nearly identical pattern of performance disruptions in the EtOH group (**Figure S1 G–L**, Table S5) though the magnitude of effects differed between treatment groups (see below).

*Distractor Condition 3*: In this challenge condition the middle stimulus in the array of 5 task stimuli served as the flashing distractor while the adjacent apertures continued to serve as the task stimuli (**Figure 4** **M–R**; Table S4). In CON rats this distractor condition induced a robust decrease in response accuracy (distractor challenge: F_1,15_ = 229.235, p<0.0001), an increase in correct response latency (distractor challenge: F_1,15_ = 50.158, p<0.0001) and a diminished capacity to discriminate between Go and NoGo trials (SI, distractor challenge: F_1,15_ = 56.786, p<0.0001). Rats chose a conservative response strategy (RI, distractor challenge: F_1,15_ = 98.872, p<0.0001) and made fewer false alarm errors (distractor challenge: F_1,15_ = 7.507, p<0.05). Premature responses did not change in CON rats during this challenge test (distractor challenge: F_1,15_ = 1.022, NS) and the challenge did not affect rats' motivation for the food reward (latency to retrieve food, distractor challenge: F_1,15_<1, NS). As with the other distractor conditions described above, this distractor induced a nearly identical pattern of performance disruptions in the EtOH group, with the exception of premature responses (**Figure S1 M–R**; Table S5).

*House light distractor – negative control challenge test*: The presence of a flashing house light during the 5C-CPT session following 1 week of abstinence resulted in only subtle disruptions in task performance for CON rats. Response accuracy during the Go trials decreased slightly (baseline: 96.86±0.41%, challenge: 92.52±1.51%, F_1,16_ = 7.723, p<0.05) and the time required to make a correct response increased (baseline: 1.06±0.02 sec, challenge: 1.18±0.03 sec, F_1,16_ = 13.635, p<0.01), indicative of moderate attentional demands imposed by the challenge test. Disruptions in 5C-CPT performance were significant albeit small in magnitude for some of the other behavioral indices as well (bias, F_1,16_ = 17.096, p<0.01; SI, F_1,16_ = 11.611, p<0.01; omissions, F_1,16_ = 11.885, p<0.01; premature responses, F_1,16_ = 5.416, p<0.05; false alarm errors, F_1,16_<1, NS). A similar profile of effects induced by this mild distractor was evident in the EtOH group (not shown), and no group differences in task performance were evident during presentation of this challenge condition (all indices, group effect, NS; group x session interaction, NS).

*Relative difficulty of distractor conditions 1–4*: Comparison of the effects of the four distractors in terms of the load imposed on the animals' attentional capacity revealed that different distractor conditions resulted in progressively impaired response accuracy (accuracy, CON, Distractor condition: F_3,45_ = 95.800, p<0.0001; Session (baseline vs. test): F_1,15_ = 192.936, p<0.0001; Distractor condition x Session: F_3,45_ = 91.581, p<0.0001; Distractor 1 vs Distractor 2, p<0.0001; Distractor 1 vs Distractor 3, p<0.0001; Distractor 1 vs House Light Distractor, NS) and poor target detection (response bias RI, CON, Distractor condition: F_3,45_ = 2.884, p<0.05; Session: F_1,15_ = 69.458, p<0.0001; Distractor type x Session: F_3,45_ = 11.868, p<0.0001; Distractor 1 vs Distractor 2, NS; Distractor 2 vs Distractor 3, p<0.01; Distractor 1 vs House Light Distractor, NS; and target sensitivity SI, CON, Distractor type: F_3,45_ = 24.725, p<0.0001; Session: F_1,15_ = 76.860, p<0.0001; Distractor type x Session: F_3,45_ = 5.888, p<0.01, Distractor 1 vs Distractor 2, NS; Distractor 1 vs Distractor 3, p<0.05; Distractor 1 vs House Light Distractor, p<0.05; Distractor 2 vs Distractor 3, p<0.05; see Table S6 for all indices). A similar profile was evident in the EtOH group (**Figure S1**; Table S7). The presence of visual distractors also consistently increased decision time as compared with baseline (correct response latency, CON, Session: F_1,15_ = 88.014, p<0.0001; EtOH, Session: F_1,30_ = 33.370, p<0.01), though the magnitude of this disruption differed between the various distractor conditions only for alcohol exposed rats (CON, Distractor type: 3_3,45_<1, NS; EtOH, Distractor type: F_3,45_ = 39.425, p<0.0001). Thus, based on within-group disruptions in response accuracy distractor conditions 1–4 may be categorized as being progressively more difficult.

*Persistent increases in motor impulsivity during the 3^rd^ week of EtOH abstinence*. An initial probe of EtOH-related disruptions in 5C-CPT was performed using distractor condition 3 during week 3 of abstinence from liquid diet. EtOH rats made more premature responses indicative of increased motor impulsivity (Figure 5A; Distractor challenge 1 vs baseline, group, F_1,30_ = 1.594, p<0.01; distractor challenge x group, F_1,30_ = 7.341, p<0.05; Table S8) and this effect persisted across 5 consecutive presentations of this challenge condition (Distractor challenge 1- 5, group, F_1,30_ = 8.052, p<0.01; group x time, F_4,120_<1, NS). As described previously, this distractor induced profound disruptions in other performance indices as compared with standard (non-challenge) 5C-CPT tests. However, no group differences were evident in regard to behavioral inhibition, response accuracy or other task indices (**Table 3**; Table S11).

**Table 3:** 5C-CPT performance indices during distractor challenges at different abstinence time-points.

Measure	Group	3 Weeks Abstinence Distractor 3	5 Weeks Abstinence Distractor 1	7 Weeks Abstinence Distractor 2	7 Weeks Abstinence Distractor 3
RI	CON	−0.43±0.04	−0.18±0.05	−0.17±0.04	−0.36±0.05
	EtOH	−0.40±0.03	−0.13±0.04	−0.14±0.04	−0.28±0.05
SI	CON	0.09±0.03	0.35±0.03	0.23±0.04	0.14±0.03
	EtOH	0.04±0.02	0.28±0.02	0.24±0.04	0.07±0.03
p[Omissions]	CON	0.47±0.03	0.39±0.03	0.25±0.01	0.40±0.03
	EtOH	0.44±0.02	0.40±0.02	0.32±0.02 (**)	0.35±0.02
Correct Response	CON	1.22±0.03	1.10±0.03	1.18±0.03	1.18±0.04
Latency (sec)	EtOH	1.21±0.03	1.10±0.02	1.17±0.02	1.20±0.04
Feeder Latency (sec)	CON	2.73±0.33	2.71±0.27	2.58±0.22	2.33±0.24
	EtOH	2.72±0.26	2.33±0.20	2.79±0.28	2.36±0.20

*Transient increases in motor impulsivity during the 5^th^ week of EtOH abstinence*. Following one month of abstinence rats were presented with distractor condition 1, which as discussed above is less challenging than the distractor presented during the earlier abstinence period. EtOH rats made significantly more premature responses than CON animals during the first test challenge (Distractor challenge 1 vs baseline, group, F_1,31_ = 4.701, p<0.05, group x distractor challenge, F_1,31_ = 4.849, p<0.05; **Figure 5D**; Table S9). This distractor induced significant disruptions in most indices of task performance relative to behavior in standard non-challenge tests, though group differences in performance were restricted to premature responses (**Table 3**; Table S12). Group differences in premature responses did not persist during subsequent presentations of this distractor challenge, although animals adapted at different rates (Distractor challenge 1–4, group, F_1,31_ = 1.808, NS, group x time, F_3,93_ = 3.734, p<0.05).

*Impaired behavioral inhibition (false alarms) during the 7^th^ week of EtOH abstinence*. EtOH rats made more false alarm errors during the first challenge test with distractor condition 2, indicative of an inability to withhold prepotent responses (**Figure 5H**, Distractor challenge 1 vs baseline, group, F_1,31_ = 4.717, p<0.05; distractor challenge: F_1,31_ = 4.741, p<0.05; group x distractor challenge, F_1,31_ = 2.225, NS;; Table S10). The effects persisted through subsequent testing (Distractor challenge 1–3, group, F_1,31_ = 9.643, p<0.01, group x time, F_2,62_<1, NS). The effect of the distractor was profound in terms of other task indices for all rats however group differences were specific to false alarm errors with no significant differences evident for other indices with the exception of errors of omission (F_1,31_ = 7.989, p<0.01; Table S13).

As described above, indices of increased motor impulsivity in EtOH rats following 4 weeks of abstinence were evident only upon initial presentation of a moderately challenging distractor (distractor 1), and these animals exhibited adaptation to this challenge in subsequent tests. To further probe the persistence of motor impulsivity following more prolonged abstinence we presented a single challenge session with the more difficult distractor 3 at the end of the 7^th^ week of abstinence (>1 month since the last presentation of this challenge). In this session CON and EtOH rats had similar profiles of premature responses (**Figure 4J**; Distractor challenge 6 vs baseline, group, F_1,30_ = 1.525, NS, group x distractor challenge, F_1,30_ = 1.947, NS). No other group differences were evident.

## Discussion

The present experiments provide evidence of increased behavioral disinhibition during protracted abstinence from chronic intermittent EtOH consumption. Using novel behavioral protocols involving attentionally-demanding distractors, we show that EtOH treated rats are impaired in terms of both motor impulsivity (indexed by premature responding) and behavioral restraint (indexed by false alarm errors) and that deficits in impulse control persist up to 7 weeks into abstinence. These disruptions were most evident during challenge sessions requiring suppression of irrelevant prepotent response information [38], consistent with clinical research observations in people with alcohol use disorders [13], [23], [24], [39]. These results confirm prior evidence of increased motor impulsivity in the 5-CSRTT during protracted EtOH abstinence [27], [28] and extend these findings into the domain of behavioral restraint.

Evidence of cognitive and behavioral aberrations among persons with alcohol use disorders during protracted abstinence is highly variable, and not all patients exhibit increased impulsivity. Human laboratory studies indicate a strong genetic component in the impulsive phenotype observed in alcoholics [40] and this may confer vulnerability to alcohol use disorders. However, evidence of increased cognitive impairment following multiple detoxifications [16], [41] and progressive recovery of brain structure and function with prolonged abstinence [2], [42], [43] suggests that alcohol exposure itself might impact impulsive behavior. The present rodent data provide further support for this latter possibility by demonstrating that chronic alcohol exposure induces persistent increases in impulsive action relative to performance indices gathered prior to alcohol exposure. Collectively, these findings raise the possibility that impaired impulse control may be both a vulnerability factor for developing problematic drinking and also a consequence of prolonged, heavy alcohol consumption.

Correlation of the progression of abstinence-related symptomatology between alcohol dependent humans and rodents is imprecise and poorly characterized. However, the present study provides several novel findings that appear to align with clinical research observations in persons with alcohol use disorders. For example, rats with a history of chronic intermittent EtOH consumption exhibit deficient behavioral restraint as indexed by increased false alarm errors during NoGo trials both during early abstinence (1-2d, standard 5C-CPT testing) and 7 weeks into abstinence (distractor challenge tests). The early abstinence data are in line with clinical research evidence demonstrating similar impairments in behavioral restraint during CPT performance in alcoholics tested 1-4 weeks into abstinence [14]. Interestingly, both EtOH-exposed rats and alcoholic patients exhibit decreased sensitivity to the Go and NoGo targets (e.g. decreased SI). In the present study, the SI deficit during early abstinence derived from excessive false alarm errors without significant alterations in hit rate (correct Go trial responses), suggesting that EtOH-exposed rats have a specific impairment in behavioral restraint during NoGo target presentation. EtOH-related increases in false alarm errors committed during standard 5C-CPT sessions resolved with abstinence (>7d) and group differences in false alarm responding were not evident during distractor challenge tests conducted at 3 and 5 weeks of abstinence, despite the fact that one of these distractor conditions significantly increased false alarm responding relative to standard 5C-CPT conditions in both EtOH and CON animals (distractor 1; **Figure 4B** **and Figure S1B**). However, significant deficits in behavioral restraint re-emerged in EtOH-exposed rats at later stages of abstinence (7 weeks) during challenge tests employing a distractor condition that did not induce *per se* increases in false alarm responding (distractor condition 2; **Figures 4H and S** **1H**).

A somewhat distinct profile was evident for increased impulsive action during protracted EtOH abstinence. Significant increases in premature responding were evident in EtOH-exposed rats during distractor challenge tests conducted at 3 and 5 weeks of abstinence, and this was more pronounced in the context of distractor condition 3 vs. distractor condition 1 (**Figure 5A, D**). Based on evidence that distractor 3 presents greater cognitive challenge vs. distractor 1 (**Figures 4** and **S1**) these findings suggest that neural mechanisms that normally constrain impulsive action under conditions of enhanced cognitive load are impaired following 3–5 weeks of abstinence from chronic EtOH exposure. These results are consistent with prior observations in rats and mice [27], [28]. Collectively, these observations raise the possibility that increased impulsive action (premature responding) persists up to 5 weeks of abstinence, while impaired behavioral restraint (false alarm responding) is transiently evident during early abstinence and re-emerges at relatively later abstinence stages (7 weeks). Interestingly, these results align with a meta analysis of clinical research in alcoholics demonstrating a progressive increase in indices of impulsivity over a long period of abstinence (1–12 months) before finally declining back toward baseline [2]. Further study is necessary to provide a better correlation of the temporal progression of abstinence-related effects between humans (in the range of days to years) and rodents (typically evaluated over the course of days to months). Further studies are also warranted to characterize the potential influence of sequencing effects in testing the various distractor conditions, and to further investigate the substantial delay (7w) in the emergence of impaired behavioral restraint during alcohol abstinence.

The 5C-CPT offers the benefit of indexing both impulsive action (premature responding) and behavioral disinhibition (false alarms) simultaneously, and this may be advantageous in the elucidation of the neural mechanisms that mediate/modulate these aspects of motor impulsivity. Lesions of the infralimbic and orbital frontal cortices significantly increase premature responses in the 5-CSRTT [44] but do not disrupt the capacity to restrain responses to NoGo stimuli [45], [46]. Accordingly, in addition to indexing temporal changes in impulse control during abstinence the distinct behavioral profiles elicited by the various distractor conditions may reflect differential engagement of networks involving the infralimbic and/or orbitofrontal cortex, both of which are damaged in the alcoholic brain [47]. Our results thus add to a growing body of evidence that impulsivity is a multi-faceted construct that relies on several different underlying circuits [36]. Serotonin signaling is known to influence the constraint of both premature responses and false alarm errors [22], [48]. As such the present observations raise the possibility that persistent deficits in cortical 5-HT signaling, perhaps 5-HT_2A_ signaling in particular [49]–[51], contribute to deficient impulse control during protracted EtOH abstinence. In this regard it is interesting that 5-HT_2A_ polymorphisms are associated with impulsive traits and suicide in alcoholics [52] and may contribute to familial alcoholism [53]. However, the long-term effects of chronic EtOH on cortical neurochemistry are not well characterized and more studies are necessary to explore these hypotheses.

EtOH-related disruptions in 5C-CPT performance were specific to facets of impulsivity and in general did not extend to the domain of selective attention (group differences in vigilance at the 3 h abstinence time-point notwithstanding). Rats in both groups maintained similar response accuracy in Go trials and did not differ in decision time (correct response latency), the ability to attend to trials (omissions) or the motivation to perform the task (latency to retrieve the food). Although performance in the attentional capacity/vigilance domain was strongly disrupted for both groups during distractor challenges, CON and EtOH rats maintained similar levels of responding. These data suggest that chronic intermittent EtOH exposure results in relatively specific impairments in motor impulsivity rather than more broad disruptions in various facets of executive function including attentional capacity [23].

Distractors have been successfully employed to reveal impaired cognitive function in humans [54], and in rodents white noise bursts have been commonly employed as distractors in 5-CSRTT tests [31], [50], [55]. The present experiments employed LED distractors to create a cognitively challenging context for unmasking EtOH-related disruptions in behavioral inhibition. Placement of the LED distractors progressively closer to the 5C-CPT stimulus panel resulted in progressively greater disruptions in task performance including deficits in response accuracy (**Figures 4** **and S1, panels A, G, M**), increased correct response latency (**Figures 4** **and S1, panels F, L, R**) and decreased target detection (**Figures 4** **and S1, panels C, I, O**). Interestingly, while EtOH-related impairments in motor impulsivity were evident during tests with unfamiliar, cognitively challenging distractors (**Figure 5**), group differences in 5C-CPT performance were not evident in tests using familiar, highly-trained task parameters or upon presentation of a mild distractor (blinking house light) that did not elicit robust performance deficits in either EtOH-treated or control subjects. This is consistent with clinical research evidence that detoxified alcoholics can sustain control-levels of cognitive performance in simple tasks through recruitment of neural networks that are not normally engaged during task performance [25], [56], though this “strategy” is insufficient to sustain normal performance in difficult tasks involving cognitive interference [13], [25], [57].

In summary, the present results provide novel evidence of impaired behavioral restraint and confirmation of increased motor impulsivity during 7 weeks of protracted abstinence from chronic intermittent EtOH exposure. These findings are consistent with behavioral clinical research studies and evidence of long-term disruptions in frontal cortical structure and function in persons with alcohol use disorders [47], In light of evidence that increased impulsivity during alcohol abstinence is a predictor of relapse [4], [58] the behavioral models described here may provide a useful platform for characterizing the neural mechanisms underlying EtOH-related impulse disorders and for identifying therapeutic approaches for the prolongation of alcohol abstinence and prevention of relapse.

## Supporting Information

Figure S1 **Characterization of the effects of visual distractors on 5C-CPT performance by EtOH-exposed rats.** In an effort to increase the cognitive load of the 5C-CPT a series of challenge tests were performed in which irrelevant prepotent visual distractors (LEDs) were illuminated in tandem with presentation of the Go and NoGo visual stimuli. Three challenge conditions were evaluated, each distinguished by a different placement of the LED distractor relative to the 5C-CPT stimulus panel (see text for details). Data shown are from EtOH-exposed rats (n = 16) to demonstrate the effects of each distractor condition on 5C-CPT performance. Group comparisons (EtOH vs. CON) under each distractor condition are shown in Figure 5 in the main text. All distractors reduced response accuracy (panel **A, G, M**; distractor 1, session: F_1,15_ = 13.818, p<0.01; distractor 2, session: F_1,15_ = 72.430, p<0.0001; distractor 3, session: F_1,15_ = 410.663, p<0.0001) and increased the latency to correct response (panel **F, L, R**; distractor 1, session: F_1,15_ = 13.818, p<0.01; distractor 2, session: F_1,15_ = 26.757, p<0.0001; distractor 3, session: F_1,15_ = 5.727, p<0.05) indicative of increased cognitive load. In the presence of distractors rats were less able to discriminate between Go and NoGo signals (panel **C, I, O**; distractor 1, session: F_1,15_ = 13.818, p<0.01; distractor 2, session: F_1,15_ = 30.632, p<0.0001; distractor 3, session: F_1,15_ = 130.491, p<0.0001) and chose a conservative response strategy (panel **D, J, P**; distractor 1, session: F_1,15_ = 13.818, p<0.01; distractor 2, session: F_1,15_ = 29.818, p<0.0001; distractor 3, session: F_1,15_ = 67.776, p<0.0001). The distractor effects described above were similar to those observed in control rats (see Figure 4 for comparison). However, in contrast to control rats distractor condition 3 induced significant increases in premature responses in EtOH-exposed rats (panel **E**, **K**, **Q**; distractor 1, session: F_1,15_ = 13.818, p<0.01; distractor 2, session: F_1,15_ = 2.665, NS; distractor 3, session: F_1,15_ = 6.791, p<0.05). Relative effects of each distractor condition on 5C-CPT performance was evaluated by within-subject ANOVA comparison of baseline performance under familiar task conditions (dark grey bars) and performance during the distractor challenge test (light grey bars). Significant effects are denoted by # p<0.05; ## p<0.01; ### p<0.001 (1-way ANOVA).
(PDF)

Table S1 **Results of statistical tests evaluating group differences in 5C - CPT performance during acute abstinence from chronic intermittent EtOH exposure (associated with** **Figure 2** **).** 1-way ANOVA with group (CON, EtOH) as between subjects factor was conducted for each task parameter independently to assess group differences in 5C-CPT performance during acute abstinence.
(PDF)

Table S2 **Results of statistical tests evaluating group differences in 5C - CPT performance during the first week of abstinence from chronic intermittent EtOH exposure (associated with** **Figure 3** **).** A 2-way ANOVA with group (CON, EtOH) as the between subjects factor and time (d1- d5 of abstinence) as the within subjects factor was used to evaluate the effects of alcohol exposure on task performance during the first week of abstinence.
(PDF)

Table S3 **Results of statistical tests evaluating group differences in 5C - CPT performance during prolonged abstinence (associated with** **Table 2** **).** Repeated measures ANOVA with group (EtOH, CON) as the between-subjects factor and abstinence time points (as defined in Table 2) as the within-subjects factor was used to evaluate 5C-CPT performance during prolonged abstinence.
(PDF)

Table S4 **Results of statistical tests evaluating changes in 5C - CPT performance in CONTROL animals following initial presentation of each distractor (associated with** **Figure 4** **).** The effects of distractor challenges were probed using 1-way ANOVA with test condition (baseline (the average of two sessions immediately prior to distractor test), distractor challenge (first presentation)) as the within-subjects factor.
(PDF)

Table S5 **Results of statistical tests evaluating changes in 5C - CPT performance in EtOH animals following initial presentation of each distractor (associated with Figure S1).** The effects of distractor challenges were probed using 1-way ANOVA with test condition (baseline (the average of two sessions immediately prior to distractor test), distractor challenge (first presentation)) as the within-subjects factor.
(PDF)

Table S6 **Results of statistical tests comparing the effects of the three distractors and the house light test on 5C-CPT performance in CONTROL animals (associated with** **Figure 4** **).** The comparative effects of the 4 challenge tests on behavior were evaluated using repeated measures ANOVA with test condition (baseline, distractor challenge) and distractor type (conditions 1, 2, 3 and house light) as within-subjects factors.
(PDF)

Table S7 **Results of statistical tests comparing the effects of the three distractors and the house light test on 5C-CPT performance in EtOH animals (associated with Figure S1).** The comparative effects of the 4 challenge tests on behavior were evaluated using repeated measures ANOVA with test condition (baseline, distractor challenge) and distractor type (conditions 1, 2, 3 and house light) as within-subjects factors.
(PDF)

Table S8 **Results of statistical tests evaluating group differences in response to the first presentation of Distractor 3 (associated with** **Figure 5** **, panel A–C).** Group differences were evaluated using 2- way mixed ANOVA with group (CON, EtOH) as a between – subjects factor and test condition (baseline, first distractor challenge) as the within-subjects factor.
(PDF)

Table S9 **Results of statistical tests evaluating group differences in response to the first presentation of Distractor 1 (associated with** **Figure 5** **, panel D–F).** Group differences were evaluated using 2- way mixed ANOVA with group (CON, EtOH) as a between – subjects factor and test condition (baseline, first distractor challenge) as the within-subjects factor.
(PDF)

Table S10 **Results of statistical tests evaluating group differences in response to the first presentation of Distractor 2 (associated with** **Figure 5** **, panel G–I).** Group differences were evaluated using 2 - way mixed ANOVA with group (CON, EtOH) as a between – subjects factor and test condition (baseline, first distractor challenge) as the within-subjects factor.
(PDF)

Table S11 **Results of statistical tests evaluating group differences in response to the repeated presentation of Distractor 3.** Group differences in response to repeated presentations of Distractor 3 were evaluated using 2 - way mixed ANOVA with group (CON, EtOH) as a between – subjects factor and time (challenge 1–5) as the within-subjects factor.
(PDF)

Table S12 **Results of statistical tests evaluating group differences in response to repeated presentation of Distractor 1.** Group differences in response to repeated presentation of Distractor 1 were evaluated using 2- way mixed ANOVA with group (CON, EtOH) as a between – subjects factor and time (challenge 1–4) as the within-subjects factor.
(PDF)

Table S13 **Results of statistical tests evaluating group differences in response to repeated presentation of Distractor 2.** Group differences in response to repeated presentation of Distractor 2 were evaluated using 2- way mixed ANOVA with group (CON, EtOH) as a between – subjects factor and time (challenge 1–3) as the within-subjects factor.
(PDF)

Data S1 (XLSX)
