# Supplementary material for: Impaired Response Inhibition in the Rat 5 Choice Continuous Performance Task during Protracted Abstinence from Chronic Alcohol Consumption
Source: PLoS One. 2014 Oct 15;9(10):e109948. doi: 10.1371/journal.pone.0109948 (PMC4198178; doi:10.1371/journal.pone.0109948)
Supplement: Figure S1 — Characterization of the effects of visual distractors on 5C-CPT performance by EtOH-exposed rats. In an effort to increase the cognitive load of the 5C-CPT a series of challenge tests were performed in which irrelevant prepotent visual distractors (LEDs) were illuminated in tandem with presentation of the Go and NoGo visual stimuli. Three challenge conditions were evaluated, each distinguished by a different placement of the LED distractor relative to the 5C-CPT stimulus panel (see text for details). Data shown are from EtOH-exposed rats (n = 16) to demonstrate the effects of each distractor condition on 5C-CPT performance. Group comparisons (EtOH vs. CON) under each distractor condition are shown in Figure 5 in the main text. All distractors reduced response accuracy (panel A, G, M; distractor 1, session: F1,15 = 13.818, p<0.01; distractor 2, session: F1,15 = 72.430, p<0.0001; distractor 3, session: F1,15 = 410.663, p<0.0001) and increased the latency to correct response (panel F, L, R; distractor 1, session: F1,15 = 13.818, p<0.01; distractor 2, session: F1,15 = 26.757, p<0.0001; distractor 3, session: F1,15 = 5.727, p<0.05) indicative of increased cognitive load. In the presence of distractors rats were less able to discriminate between Go and NoGo signals (panel C, I, O; distractor 1, session: F1,15 = 13.818, p<0.01; distractor 2, session: F1,15 = 30.632, p<0.0001; distractor 3, session: F1,15 = 130.491, p<0.0001) and chose a conservative response strategy (panel D, J, P; distractor 1, session: F1,15 = 13.818, p<0.01; distractor 2, session: F1,15 = 29.818, p<0.0001; distractor 3, session: F1,15 = 67.776, p<0.0001). The distractor effects described above were similar to those observed in control rats (see Figure 4 for comparison). However, in contrast to control rats distractor condition 3 induced significant increases in premature responses in EtOH-exposed rats (panel E, K, Q; distractor 1, session: F1,15 = 13.818, p<0.01; distractor 2, session: F1,15 = 2.665, N [file pone.0109948.s001.pdf]

Supplementary Figure S1. Characterization of the effects of visual distractors on 5C-CPT performance by EtOH-exposed rats. In an effort to increase the cognitive load of the 5C-CPT a series of challenge tests were performed in which irrelevant prepotent visual distractors (LEDs) were illuminated in tandem with presentation of the Go and NoGo visual stimuli. Three challenge conditions were evaluated, each distinguished by a different placement of the LED distractor relative to the 5C-CPT stimulus panel (see text for details). Data shown are from EtOH-exposed rats (n = 16) to demonstrate the effects of each distractor condition on 5C-CPT performance. Group comparisons (EtOH vs. CON) under each distractor condition are shown in Figure 5 in the main text. All distractors reduced response accuracy (panel **A, G, M**; distractor 1, session: $F_{1,15}$=13.818, p<0.01; distractor 2, session: $F_{1,15}$=72.430, p<0.0001; distractor 3, session: $F_{1,15}$=410.663, p<0.0001) and increased the latency to correct response (panel **F, L, R**; distractor 1, session: $F_{1,15}$=13.818, p<0.01; distractor 2, session: $F_{1,15}$=26.757, p<0.0001; distractor 3, session: $F_{1,15}$=5.727, p<0.05) indicative of increased cognitive load. In the presence of distractors rats were less able to discriminate between Go and NoGo signals (panel **C, I, O;** distractor 1, session: $F_{1,15}$=13.818, p<0.01; distractor 2, session: $F_{1,15}$=30.632, p<0.0001; distractor 3, session: $F_{1,15}$=130.491, p<0.0001) and chose a conservative response strategy (panel **D, J, P;** distractor 1, session: $F_{1,15}$=13.818, p<0.01; distractor 2, session: $F_{1,15}$=29.818, p<0.0001; distractor 3, session: $F_{1,15}$=67.776, p<0.0001). The distractor effects described above were similar to those observed in control rats (see Figure 4 for comparison). However, in contrast to control rats distractor condition 3 induced significant increases in premature responses in EtOH-exposed rats (panel **E**, **K**, **Q**; distractor 1, session: $F_{1,15}$=13.818, p<0.01; distractor 2, session: $F_{1,15}$=2.665, NS; distractor 3, session: $F_{1,15}$=6.791, p<0.05). Relative effects of each distractor condition on 5C-CPT performance was evaluated by within-subject ANOVA comparison of baseline performance under familiar task conditions (dark grey bars) and performance during the distractor challenge test (light grey bars). Significant effects are denoted by # p < 0.05; ## p< 0.01; ### p < 0.001 (1-way ANOVA).

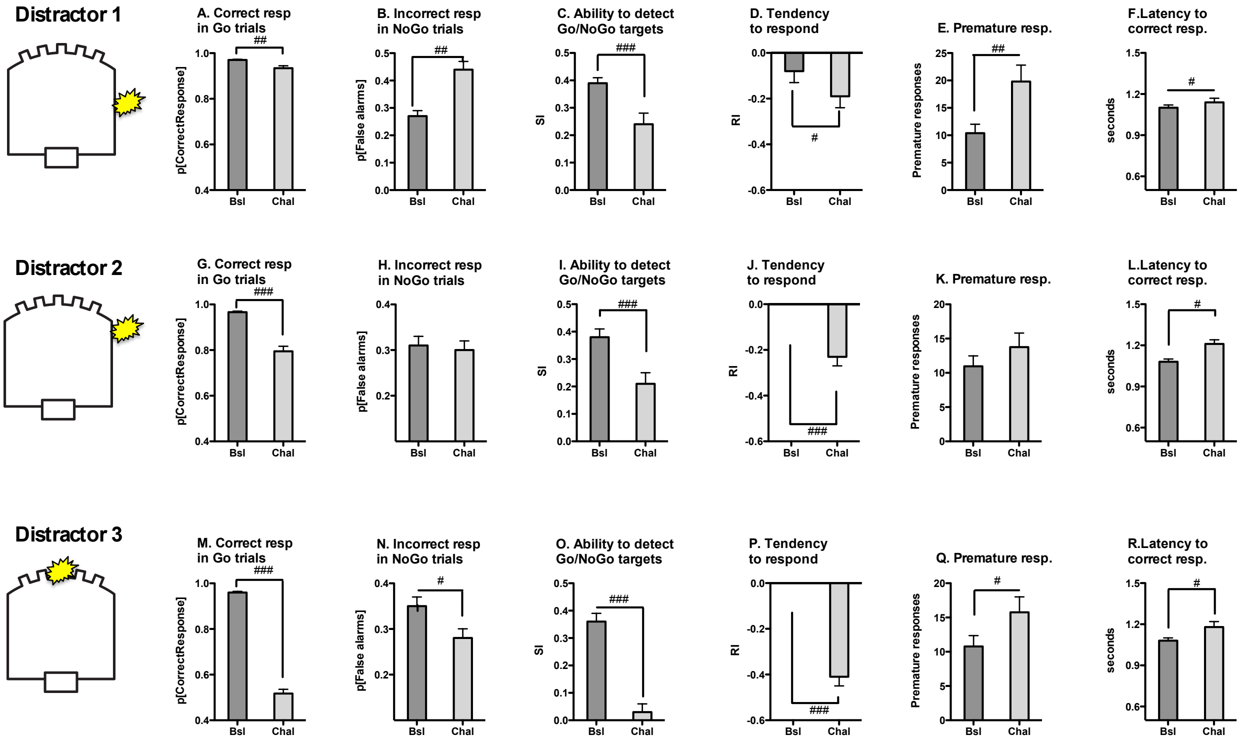

Distractor 1
A. Correct resp in Go trials
B. Incorrect resp in NoGo trials
C. Ability to detect Go/NoGo targets
D. Tendency to respond
E. Premature resp.
F.Latency to correct resp.
Distractor 2
G. Correct resp in Go trials
H. Incorrect resp in NoGo trials
I. Ability to detect Go/NoGo targets
J. Tendency to respond
K. Premature resp.
L.Latency to correct resp.
Distractor 3
M. Correct resp in Go trials
N. Incorrect resp in NoGo trials
O. Ability to detect Go/NoGo targets
P. Tendency to respond
Q. Premature resp.
R.Latency to correct resp.
p[CorrectResponse]
p[False alarms]
SI
RI
Premature responses
seconds
Bsl
Chal
