# Supplementary material for: Impaired Response Inhibition in the Rat 5 Choice Continuous Performance Task during Protracted Abstinence from Chronic Alcohol Consumption
Source: PLoS One. 2014 Oct 15;9(10):e109948. doi: 10.1371/journal.pone.0109948 (PMC4198178; doi:10.1371/journal.pone.0109948)
Supplement: Table S1 — Results of statistical tests evaluating group differences in 5C - CPT performance during acute abstinence from chronic intermittent EtOH exposure (associated with Figure 2 ). 1-way ANOVA with group (CON, EtOH) as between subjects factor was conducted for each task parameter independently to assess group differences in 5C-CPT performance during acute abstinence. (PDF) [file pone.0109948.s002.pdf]

Supplementary Table S1. Results of statistical tests evaluating group differences in 5C - CPT performance during acute abstinence from chronic intermittent EtOH exposure (associated with Figure 2). 1-way ANOVA with Group (CON, EtOH) as between subjects factor was conducted for each task parameter independently to assess group differences in 5C-CPT performance during acute abstinence.

5C-CPT measure	$F_{(1,30)}$	p
**Accuracy**	2.268	NS
**Correct response latency**	12.088	<0.01 (**)
**False Alarm latency**	0.003	NS
**Omissions**	22.604	<0.0001(***)
**Feeder latency**	0.598	NS
**Premature resp.**	2.864	NS
**Perseverative resp.**	0.109	NS
**False alarms**	3.257	NS
**Sensitivity**	9.101	<0.01(**)
**Bias**	16.732	<0.0001 (***)
