# Supplementary material for: Impaired Response Inhibition in the Rat 5 Choice Continuous Performance Task during Protracted Abstinence from Chronic Alcohol Consumption
Source: PLoS One. 2014 Oct 15;9(10):e109948. doi: 10.1371/journal.pone.0109948 (PMC4198178; doi:10.1371/journal.pone.0109948)
Supplement: Table S2 — Results of statistical tests evaluating group differences in 5C - CPT performance during the first week of abstinence from chronic intermittent EtOH exposure (associated with Figure 3 ). A 2-way ANOVA with group (CON, EtOH) as the between subjects factor and time (d1- d5 of abstinence) as the within subjects factor was used to evaluate the effects of alcohol exposure on task performance during the first week of abstinence. (PDF) [file pone.0109948.s003.pdf]

Supplementary Table S2. Results of statistical tests evaluating group differences in 5C - CPT performance during the first week of abstinence from chronic intermittent EtOH exposure (associated with Figure 3). A 2-way ANOVA with group (CON, EtOH) as the between subjects factor and time (d1- d5 of abstinence) as the within subjects factor was used to evaluate the effects of alcohol exposure on task performance during the first week of abstinence.

5C-CPT measure	Group $F_{(1,31)}$	Group p	Time $F_{(3,93)}$	Time p	Time x Group $F_{(3,93)}$	Time x Group p
**Accuracy**	0.835	NS	1.109	NS	1.400	NS
**Correct response latency**	2.452	NS	0.908	NS	1.917	NS
**Omissions**	0.647	NS	3.241	<0.05(*)	0.440	NS
**Feeder latency**	1.439	NS	4.024	<0.05 (*)	0.691	NS
**Premature resp.**	0.738	NS	2.495	NS	1.050	NS
**Perseverative resp.**	0.195	NS	1.643	NS	2.065	NS
**False alarms**	5.712	<0.05 (*)	3.238	<0.05(*)	0.664	NS
**Sensitivity**	6.818	<0.05(*)	5.461	<0.01(**)	0.015	NS
**Bias**	0.088	NS	2.755	<0.05 (*)	0.155	NS
