# Supplementary material for: Impaired Response Inhibition in the Rat 5 Choice Continuous Performance Task during Protracted Abstinence from Chronic Alcohol Consumption
Source: PLoS One. 2014 Oct 15;9(10):e109948. doi: 10.1371/journal.pone.0109948 (PMC4198178; doi:10.1371/journal.pone.0109948)
Supplement: Table S3 — Results of statistical tests evaluating group differences in 5C - CPT performance during prolonged abstinence (associated with Table 2 ). Repeated measures ANOVA with group (EtOH, CON) as the between-subjects factor and abstinence time points (as defined in Table 2) as the within-subjects factor was used to evaluate 5C-CPT performance during prolonged abstinence. (PDF) [file pone.0109948.s004.pdf]

Supplementary Table S3. Results of statistical tests evaluating group differences in 5C - CPT performance during prolonged abstinence (associated with Table 2). Repeated measures ANOVA with group (EtOH, CON) as the between-subjects factor and abstinence time points (as defined in Table 2) as the within-subjects factor was used to evaluate 5C-CPT performance during prolonged abstinence.

5C-CPT measure	Group $F_{(1,31)}$	Group p	Time $F_{(3,93)}$	Time p	Time x Group $F_{(3,93)}$	Time x Group p
**Accuracy**	2.191	NS	1.309	NS	1.409	NS
**Correct response latency**	2.396	NS	0.571	NS	0.790	NS
**Omissions**	0.844	NS	5.886	<0.01(**)	2.068	NS
**Feeder latency**	1.854	NS	3.221	<0.05 (*)	0.518	NS
**Premature resp.**	1.020	NS	2.218	NS	0.781	NS
**Perseverative resp.**	0.430	NS	3.145	<0.05(*)	0.930	NS
**False alarms**	0.084	NS	13.859	<0.001(***)	2.020	NS
**Sensitivity**	2.188	NS	6.753	<0.01(***)	1.925	NS
**Bias**	0.696	NS	8.073	<0.001(***)	1.957	NS
