# Supplementary material for: Impaired Response Inhibition in the Rat 5 Choice Continuous Performance Task during Protracted Abstinence from Chronic Alcohol Consumption
Source: PLoS One. 2014 Oct 15;9(10):e109948. doi: 10.1371/journal.pone.0109948 (PMC4198178; doi:10.1371/journal.pone.0109948)
Supplement: Table S4 — Results of statistical tests evaluating changes in 5C - CPT performance in CONTROL animals following initial presentation of each distractor (associated with Figure 4 ). The effects of distractor challenges were probed using 1-way ANOVA with test condition (baseline (the average of two sessions immediately prior to distractor test), distractor challenge (first presentation)) as the within-subjects factor. (PDF) [file pone.0109948.s005.pdf]

Supplementary Table S4. Results of statistical tests evaluating changes in 5C - CPT performance in CONTROL animals following initial presentation of each distractor (associated with Figure 4). The effects of distractor challenges were probed using 1-way ANOVA with test condition (baseline (the average of two sessions immediately prior to distractor test), distractor challenge (first presentation)) as the within-subjects factor.

5C-CPT measure	Distractor 1 Test $F_{(1,16)}$	Distractor 1 Test p	Distractor 2 Test $F_{(1,16)}$	Distractor 2 Test p	Distractor 3 Test $F_{(1,15)}$	Distractor 3 Test p
**Accuracy**	6.957	<0.05 (*)	95.628	<0.001(***)	229.235	<0.001(***)
**Correct response latency**	19.316	<0.001(***)	27.070	<0.001(***)	50.158	<0.001(***)
**Omissions**	44.993	<0.001(***)	2.241	NS	29.140	<0.001(***)
**Feeder latency**	1.390	NS	1.600	NS	0.572	NS
**Premature resp.**	5.111	<0.05 (*)	2.602	NS	1.022	NS
**Perseverative resp.**	0.249	NS	6.786	<0.05(*)	77.086	<0.001(***)
**False alarms**	26.492	<0.001(***)	8.811	<0.01(**)	7.507	<0.05(*)
**Sensitivity**	25.638	<0.001(***)	28.492	<0.000(***)	56.786	<0.001(***)
**Bias**	16.254	<0.01(**)	23.810	<0.001(***)	98.872	<0.001(***)
