# Supplementary material for: Impaired Response Inhibition in the Rat 5 Choice Continuous Performance Task during Protracted Abstinence from Chronic Alcohol Consumption
Source: PLoS One. 2014 Oct 15;9(10):e109948. doi: 10.1371/journal.pone.0109948 (PMC4198178; doi:10.1371/journal.pone.0109948)
Supplement: Table S5 — Results of statistical tests evaluating changes in 5C - CPT performance in EtOH animals following initial presentation of each distractor (associated with Figure S1). The effects of distractor challenges were probed using 1-way ANOVA with test condition (baseline (the average of two sessions immediately prior to distractor test), distractor challenge (first presentation)) as the within-subjects factor. (PDF) [file pone.0109948.s006.pdf]

Supplementary Table S5. Results of statistical tests evaluating changes in 5C - CPT performance in EtOH animals following initial presentation of each distractor (associated with Supplementary Figure 1). The effects of distractor challenges were probed using 1-way ANOVA with test condition (baseline (the average of two sessions immediately prior to distractor test), distractor challenge (first presentation)) as the within-subjects factor.

5C-CPT measure	Distractor 1 Test	Distractor 1 Test	Distractor 2 Test	Distractor 2 Test	Distractor 3 Test	Distractor 3 Test
	$F_{(1,15)}$	p	$F_{(1,15)}$	p	$F_{(1,15)}$	p
**Accuracy**	13.818	<0.01(**)	72.430	<0.001(***)	410.663	<0.001(***)
**Correct response latency**	5.583	<0.05(*)	26.757	<0.001(***)	5.727	<0.05(*)
**Omissions**	27.572	<0.001(***)	0.177	NS	11.435	<0.01(**)
**Feeder latency**	1.304	NS	1.613	NS	0.138	NS
**Premature resp.**	14.542	<0.01(**)	2.665	NS	6.791	<0.05(*)
**Perseverative resp.**	9.996	<0.01(**)	16.762	<0.01(**)	58.970	<0.001(***)
**False alarms**	14.179	<0.01(**)	0.186	NS	5.324	<0.05(*)
**Sensitivity**	19.898	<0.001(***)	30.632	<0.001(***)	130.491	<0.001(***)
**Bias**	6.934	<0.05(*)	29.818	0.001(***)	67.776	<0.001(***)
