# Supplementary material for: Impaired Response Inhibition in the Rat 5 Choice Continuous Performance Task during Protracted Abstinence from Chronic Alcohol Consumption
Source: PLoS One. 2014 Oct 15;9(10):e109948. doi: 10.1371/journal.pone.0109948 (PMC4198178; doi:10.1371/journal.pone.0109948)
Supplement: Table S6 — Results of statistical tests comparing the effects of the three distractors and the house light test on 5C-CPT performance in CONTROL animals (associated with Figure 4 ). The comparative effects of the 4 challenge tests on behavior were evaluated using repeated measures ANOVA with test condition (baseline, distractor challenge) and distractor type (conditions 1, 2, 3 and house light) as within-subjects factors. (PDF) [file pone.0109948.s007.pdf]

Supplementary Table S6. Results of statistical tests comparing the effects of the three distractors and the house light test on 5C-CPT performance in CONTROL animals (associated with Figure 4). The comparative effects of the 4 challenge tests on behavior were evaluated using repeated measures ANOVA with test condition (baseline, distractor challenge) and distractor type (conditions 1, 2, 3 and house light) as within-subjects factors.

5C-CPT measure	Distractor Type $F_{(3,45)}$	Distractor Type p	Session $F_{(1,15)}$	Session p	Distractor Type x Session $F_{(3,45)}$	Distractor Type x Session p
**Accuracy**	95.800	<0.001(***)	192.936	<0.001(***)	91.581	<0.001(***)
**Correct response latency**	0.943	NS	88.014	<0.001(***)	2.001	NS
**Omissions**	20.779	<0.001(***)	31.918	<0.001(***)	12.546	<0.001(***)
**Feeder latency**	0.785	NS	4.990	<0.05 (*)	0.375	NS
**Premature resp.**	2.110	NS	4.410	NS	2.477	NS
**Perseverative resp.**	56.157	<0.001(***)	76.835	<0.001(***)	64.308	<0.001(***)
**False alarms**	20.576	<0.001(***)	2.531	NS	25.396	<0.001(***)
**Sensitivity**	24.725	<0.001(***)	76.860	<0.001(***)	5.888	<0.01(**)
**Bias**	2.884	<0.05	69.458	<0.001(***)	11.868	<0.001(***)
