# Supplementary material for: Impaired Response Inhibition in the Rat 5 Choice Continuous Performance Task during Protracted Abstinence from Chronic Alcohol Consumption
Source: PLoS One. 2014 Oct 15;9(10):e109948. doi: 10.1371/journal.pone.0109948 (PMC4198178; doi:10.1371/journal.pone.0109948)
Supplement: Table S7 — Results of statistical tests comparing the effects of the three distractors and the house light test on 5C-CPT performance in EtOH animals (associated with Figure S1). The comparative effects of the 4 challenge tests on behavior were evaluated using repeated measures ANOVA with test condition (baseline, distractor challenge) and distractor type (conditions 1, 2, 3 and house light) as within-subjects factors. (PDF) [file pone.0109948.s008.pdf]

Supplementary Table S7. Results of statistical tests comparing the effects of the three distractors and the house light test on 5C-CPT performance in EtOH animals (associated with Supplementary Figure 1). The comparative effects of the 4 challenge tests on behavior were evaluated using repeated measures ANOVA with test condition (baseline, distractor challenge) and distractor type (conditions 1, 2, 3 and house light) as within-subjects factors.

5C-CPT measure	Distractor Type $F_{(2,60)}$	Distractor Type p	Session $F_{(1,30)}$	Session p	Distractor Type x Session $F_{(2,60)}$	Distractor Type x Session p
**Accuracy**	143.680	<0.001(***)	368.190	<0.001(***)	156.616	<0.001(***)
**Correct response latency**	39.425	<0.001(***)	33.370	<0.001(**)	40.929	<0.001(***)
**Omissions**	8.549	<0.001(***)	26.037	<0.05(*)	4.009	<0.05(*)
**Feeder latency**	0.878	NS	2.226	NS	0.835	NS
**Premature resp.**	1.183	NS	25.364	<0.001(***)	1.550	NS
**Perseverative resp.**	56.157	<0.001(***)	76.835	<0.001(***)	64.308	<0.001(***)
**False alarms**	20.694	<0.001(***)	0.340	NS	15.032	<0.001(***)
**Sensitivity**	27.764	<0.001(***)	115.713	<0.001(***)	16.737	<0.001(***)
**Bias**	67.389	<0.001(***)	76.343	<0.001(***)	66.427	<0.001(***)
