# Supplementary material for: Impaired Response Inhibition in the Rat 5 Choice Continuous Performance Task during Protracted Abstinence from Chronic Alcohol Consumption
Source: PLoS One. 2014 Oct 15;9(10):e109948. doi: 10.1371/journal.pone.0109948 (PMC4198178; doi:10.1371/journal.pone.0109948)
Supplement: Table S8 — Results of statistical tests evaluating group differences in response to the first presentation of Distractor 3 (associated with Figure 5 , panel A–C). Group differences were evaluated using 2- way mixed ANOVA with group (CON, EtOH) as a between – subjects factor and test condition (baseline, first distractor challenge) as the within-subjects factor. (PDF) [file pone.0109948.s009.pdf]

Supplementary Table S8. Results of statistical tests evaluating group differences in response to the first presentation of Distractor 3 (associated with Figure 5, panel A - C). Group differences were evaluated using 2- way mixed ANOVA with group (CON, EtOH) as a between – subjects factor and test condition (baseline, first distractor challenge) as the within-subjects factor.

5C-CPT measure	Distractor 3 Group $F_{(1,30)}$	Distractor 3 Group p	Distractor 3 Challenge $F_{(1,30)}$	Distractor 3 Challenge p	Distractor 3 Group x challenge $F_{(1,30)}$	Distractor 3 Group x challenge p
**Accuracy**	1.535	NS	597.048	<0.001(***)	0.423	NS
**Correct response latency**	0.660	NS	38.592	<0.001(***)	5.840	<0.05(*)
**Omissions**	0.120	NS	35.435	<0.001(***)	0.311	NS
**Feeder latency**	0.031	NS	0.006	NS	0.0508	NS
**Premature resp.**	1.594	NS	2.363	NS	7.341	<0.05(*)
**Perseverative resp.**	0.051	NS	134.071	<0.001(***)	0.021	NS
**False alarms**	2.495	NS	12.264	<0.01(**)	0.021	NS
**Sensitivity**	1.246	NS	163.911	<0.001(***)	0.354	NS
**Bias**	1.910	NS	156.469	<0.001(***)	0.641	NS
