# Supplementary material for: Impaired Response Inhibition in the Rat 5 Choice Continuous Performance Task during Protracted Abstinence from Chronic Alcohol Consumption
Source: PLoS One. 2014 Oct 15;9(10):e109948. doi: 10.1371/journal.pone.0109948 (PMC4198178; doi:10.1371/journal.pone.0109948)
Supplement: Table S9 — Results of statistical tests evaluating group differences in response to the first presentation of Distractor 1 (associated with Figure 5 , panel D–F). Group differences were evaluated using 2- way mixed ANOVA with group (CON, EtOH) as a between – subjects factor and test condition (baseline, first distractor challenge) as the within-subjects factor. (PDF) [file pone.0109948.s010.pdf]

Supplementary Table S9. Results of statistical tests evaluating group differences in response to the first presentation of Distractor 1 (associated with Figure 5, panel D - F). Group differences were evaluated using 2 - way mixed ANOVA with group (CON, EtOH) as a between – subjects factor and test condition (baseline, first distractor challenge) as the within-subjects factor.

5C-CPT measure	Distractor 1 Group $F_{(1,31)}$	Distractor 1 Group p	Distractor 1 Challenge $F_{(1,31)}$	Distractor 1 Challenge p	Distractor 1 Group x challenge $F_{(1,31)}$	Distractor 1 Group x challenge p
**Accuracy**	0.009	NS	17.434	<0.001(***)	0.038	NS
**Correct response latency**	0.732	NS	24.073	<0.001(***)	6.737	<0.05(*)
**Omissions**	0.073	NS	71.589	<0.001(***)	1.821	NS
**Feeder latency**	0.348	NS	2.655	NS	0.021	NS
**Premature resp.**	4.701	<0.05(*)	20.245	<0.001(***)	4.849	<0.05(*)
**Perseverative resp.**	1.673	NS	4.081	NS	7.232	<0.05(*)
**False alarms**	0.003	NS	39.734	<0.001(***)	1.126	NS
**Sensitivity**	0.155	NS	45.024	<0.001(***)	0.853	NS
**Bias**	1.236	NS	17.624	<0.001(***)	0.410	NS
