# Supplementary material for: Impaired Response Inhibition in the Rat 5 Choice Continuous Performance Task during Protracted Abstinence from Chronic Alcohol Consumption
Source: PLoS One. 2014 Oct 15;9(10):e109948. doi: 10.1371/journal.pone.0109948 (PMC4198178; doi:10.1371/journal.pone.0109948)
Supplement: Table S10 — Results of statistical tests evaluating group differences in response to the first presentation of Distractor 2 (associated with Figure 5 , panel G–I). Group differences were evaluated using 2 - way mixed ANOVA with group (CON, EtOH) as a between – subjects factor and test condition (baseline, first distractor challenge) as the within-subjects factor. (PDF) [file pone.0109948.s011.pdf]

Supplementary Table S10. Results of statistical tests evaluating group differences in response to the first presentation of Distractor 2 (associated with Figure 5, panel G - I). Group differences were evaluated using 2 - way mixed ANOVA with group (CON, EtOH) as a between – subjects factor and test condition (baseline, first distractor challenge) as the within-subjects factor.

5C-CPT measure	Distractor 2 Group $F_{(1,31)}$	Distractor 2 Group p	Distractor 2 Challenge $F_{(1,31)}$	Distractor 2 Challenge p	Distractor 2 Group x challenge $F_{(1,31)}$	Distractor 2 Group x challenge p
**Accuracy**	0.236	NS	165.365	<0.001(***)	0.075	NS
**Correct response latency**	0.011	NS	51.448	<0.001(***)	1.349	NS
**Omissions**	1.973	NS	0.431	NS	1.684	NS
**Feeder latency**	0.461	NS	3.194	NS	0.002	NS
**Premature resp.**	0.040	NS	5.265	<0.05(*)	0.002	NS
**Perseverative resp.**	0.002	NS	20.623	<0.001(***)	0.231	NS
**False alarms**	4.717	<0.05(*)	4.741	<0.05(*)	2.225	NS
**Sensitivity**	0.662	NS	59.314	<0.001(***)	0.260	NS
**Bias**	1.277	NS	52.871	<0.001(***)	0.026	NS
