# Supplementary material for: Impaired Response Inhibition in the Rat 5 Choice Continuous Performance Task during Protracted Abstinence from Chronic Alcohol Consumption
Source: PLoS One. 2014 Oct 15;9(10):e109948. doi: 10.1371/journal.pone.0109948 (PMC4198178; doi:10.1371/journal.pone.0109948)
Supplement: Table S11 — Results of statistical tests evaluating group differences in response to the repeated presentation of Distractor 3. Group differences in response to repeated presentations of Distractor 3 were evaluated using 2 - way mixed ANOVA with group (CON, EtOH) as a between – subjects factor and time (challenge 1–5) as the within-subjects factor. (PDF) [file pone.0109948.s012.pdf]

Supplementary Table S11. Results of statistical tests evaluating group differences in response to the repeated presentation of Distractor 3. Group differences in response to repeated presentations of Distractor 3 were evaluated using 2 - way mixed ANOVA with group (CON, EtOH) as a between – subjects factor and time (challenge 1 - 5) as the within-subjects factor.

5C-CPT measure	Distractor 3 Group $F_{(1,31)}$	Distractor 3 Group p	Distractor 3 Time $F_{(4,120)}$	Distractor 3 Time p	Distractor 3 Group x time $F_{(4,120)}$	Distractor 3 Group x time p
**Accuracy**	1.204	NS	3.184	<0.05(*)	1.298	NS
**Correct response latency**	0.035	NS	1.091	NS	1.713	NS
**Omissions**	0.606	NS	2.310	NS	0.045	NS
**Feeder latency**	0.000	NS	1.964	NS	0.619	NS
**Premature resp.**	8.052	<0.01(**)	1.404	NS	0.198	NS
**Perseverative resp.**	0.150	NS	31.384	<0.001(***)	0.225	NS
**False alarms**	1.410	NS	3.376	<0.05(*)	0.454	NS
**Sensitivity**	1.543	NS	2.290	NS	0.993	NS
**Bias**	0.464	NS	2.237	NS	0.149	NS
