# Supplementary material for: Impaired Response Inhibition in the Rat 5 Choice Continuous Performance Task during Protracted Abstinence from Chronic Alcohol Consumption
Source: PLoS One. 2014 Oct 15;9(10):e109948. doi: 10.1371/journal.pone.0109948 (PMC4198178; doi:10.1371/journal.pone.0109948)
Supplement: Table S12 — Results of statistical tests evaluating group differences in response to repeated presentation of Distractor 1. Group differences in response to repeated presentation of Distractor 1 were evaluated using 2- way mixed ANOVA with group (CON, EtOH) as a between – subjects factor and time (challenge 1–4) as the within-subjects factor. (PDF) [file pone.0109948.s013.pdf]

Supplementary Table S12. Results of statistical tests evaluating group differences in response to repeated presentation of Distractor 1. Group differences in response to repeated presentation of Distractor 1 were evaluated using 2- way mixed ANOVA with group (CON, EtOH) as a between – subjects factor and time (challenge 1 - 4) as the within-subjects factor.

5C-CPT measure	Distractor 1 Group $F_{(1,31)}$	Distractor 1 Group p	Distractor 1 Time $F_{(3,93)}$	Distractor 1 Time p	Distractor 1 Group x time $F_{(3,93)}$	Distractor 1 Group x time p
**Accuracy**	0.822	NS	3.470	<0.05(*)	0.829	NS
**Correct response latency**	0.052	NS	17.033	<0.001(***)	4.745	<0.01(**)
**Omissions**	0.047	NS	15.967	<0.001(***)	1.480	NS
**Feeder latency**	1.205	NS	3.125	<0.05(*)	0.338	NS
**Premature resp.**	1.808	NS	7.992	<0.001(***)	3.734	<0.05(*)
**Perseverative resp.**	0.693	NS	4.664	<0.01(**)	2.981	<0.05(*)
**False alarms**	0.047	NS	15.967	<0.001(***)	1.480	NS
**Sensitivity**	3.069	NS	7.468	<0.001(***)	3.011	<0.05(*)
**Bias**	0.422	NS	9.328	<0.001(***)	0.141	NS
