# Supplementary material for: Impaired Response Inhibition in the Rat 5 Choice Continuous Performance Task during Protracted Abstinence from Chronic Alcohol Consumption
Source: PLoS One. 2014 Oct 15;9(10):e109948. doi: 10.1371/journal.pone.0109948 (PMC4198178; doi:10.1371/journal.pone.0109948)
Supplement: Table S13 — Results of statistical tests evaluating group differences in response to repeated presentation of Distractor 2. Group differences in response to repeated presentation of Distractor 2 were evaluated using 2- way mixed ANOVA with group (CON, EtOH) as a between – subjects factor and time (challenge 1–3) as the within-subjects factor. (PDF) [file pone.0109948.s014.pdf]

Supplementary Table S13. Results of statistical tests evaluating group differences in response to repeated presentation of Distractor 2. Group differences in response to repeated presentation of Distractor 2 were evaluated using 2- way mixed ANOVA with group (CON, EtOH) as a between – subjects factor and time (challenge 1 - 3) as the within-subjects factor

5C-CPT measure	Distractor 2 Group $F_{(1,31)}$	Distractor 2 Group p	Distractor 2 Time $F_{(2,62)}$	Distractor 2 Time p	Distractor 2 Group x time $F_{(2,62)}$	Distractor 2 Group x time p
**Accuracy**	0.005	NS	16.716	<0.001(***)	0.909	NS
**Correct response latency**	0.193	NS	4.614	<0.05(*)	0.474	NS
**Omissions**	7.989	<0.01(**)	4.419	<0.05(*)	0.414	NS
**Feeder latency**	0.387	NS	1.813	NS	0.558	NS
**Premature resp.**	0.435	NS	3.444	<0.05(*)	0.187	NS
**Perseverative resp.**	0.071	NS	1.042	NS	0.000	NS
**False alarms**	9.643	<0.01(**)	3.643	<0.05(*)	0.359	NS
**Sensitivity**	1.580	NS	4.922	<0.05(*)	1.199	NS
**Bias**	0.288	NS	17.872	<0.001(***)	0.188	NS
